# Book of Abstracts

**DOI:** 10.1080/24740527.2023.2251872

**Published:** 2023-12-22

**Keynote**

**Fibromyalgia: Attempts at uncovering the pathophysiology**

Claudia Sommer

Professor of Neurology, University of Würzburg, Germany

**Abstract**: Fibromyalgia syndrome (FMS) is characterized by chronic widespread pain as well as various additional symptoms and affects 2-4% of the population. The understanding of its etiology and pathogenesis is still insufficient, and many questions remain open. Central sensitization, infections, inflammatory mediators, hormones, neurotransmitters and many other factors have been discussed in the pathophysiology. In recent years, small nerve fiber pathology has been discovered in patients with FMS. We now have a large dataset of women with FMS who underwent neurological examination, questionnaire assessment, neurophysiology, and small fiber tests: skin punch biopsy, corneal confocal microscopy, microneurography, quantitative sensory testing including C-tactile afferents, and pain-related evoked potentials. We found that intraepidermal nerve fiber density (IENFD) was reduced at any biopsy site in 63% of FMS patients. There were four distinct patterns of skin innervation: normal, distally reduced, proximally reduced proximal, and both distally and proximally reduce. Microneurography revealed altered activity-dependent slowing of conduction velocity, spontaneous activity, and mechanical sensitization in FMS patients. Patients had elevated warm detection thresholds, impaired C-tactile afferents and reduced amplitudes of pain-related evoked potentials compared to controls. Compared to patients with normal skin innervation, those with generalized IENFD reduction had more stabbing pain and paresthesias, higher pain intensity and impairment due to pain, higher disease burden, and more anxiety. Using magnetic resonance imaging (MRI), we also found that the patients with generalized IENFD reduction had more pronounced CNS alterations. These findings add to the discussion on peripheral and central components of the pain in FMS.

**At the end of this presentation, the participant will be able to**:

- Cite and apply the current criteria for fibromyalgia syndrome.
- Name and critically evaluate current theories on the pathophysiology of fibromyalgia.
- Discuss peripheral and central components of the fibromyalgia syndrome.

**Plenary**

**Neuroimmune interactions for the induction and resolution of pain**

Ru -Rong Ji^a,b^

^a^Center for Translational Pain Center (CTPM), ^b^Duke Medical Center, Durham, North Carolina, United States

**Abstract**: Pain research in the past two decades has demonstrated important roles of non-neuronal cells such as immune cells and glial cells in the pathogenesis of pain via neuroimmune interactions. I will review several lines of evidence that immune and glial cells, such as macrophages, microglia, astrocytes, and satellite glial cells can drive chronic pain via inflammatory cytokines and chemokines, which are potent neuromodulators. Accumulating evidence also suggests that immune cells and glial cells further regulate the resolution of pain by producing anti-inflammatory and pro-resolution mediators. It is increasingly appreciated that disrupted resolution of acute pain will lead to the development of chronic pain. Notably, specialized pro-resolving mediators (SPMs) including resolvins, derived from omega-3 unsaturated fatty acids, exhibit potent analgesic actions via neuroimmune modulation. Recent studies also suggest that neuromodulation, such as spinal cord stimulation and vagus nerve stimulation can effectively control neuroinflammation by stimulating the pro-resolution pathways. Surprisingly, immune activation may promote pain resolution under the immune suppressive conditions. Finally, I will discuss several novel approaches that may promote pain resolution via neuroimmune regulation.

**At the end of this presentation, participants will be able to**:

- Describe the role of glial and immune cells in the induction and resolution of pain.

- Review evidence that pro-resolution mediators, such as resolvins, can alleviate pain.
- Discuss how neuromodulation controls pain via neuroimmune interactions.

**Plenary**

**Rethinking equity, diversity, inclusion and accessibility in 2023**

Gaynor Watson-Creed

Associate Dean of Serving and Engaging Society for Dalhousie University’s Faculty of Medicine, Halifax, Nova Scotia, Canada

**Abstract:** Anti-oppressive practice is an approach to the practice of medicine and other health professions that challenges practitioners to examine their own identities, intersections of those identities, and resulting power imbalances that may negatively impact patient care, teaching, and collegial relationships. In this session, attendees will receive an overview of why medicine is changing – rapidly – towards anti-oppressive practice, what anti-oppression is, and implications for pain medicine. Other concepts explored in this session will include the topics of white fragility and institutional betrayal.

**At the end of this presentation, the participant will be able to**:

- Recognize how practice and profession of medicine has complicit in upholding forms of oppression, using racism as the primary example.
- Explore the concept of anti-oppressive practice, including the concepts of identity, intersectionality, and power.
- Reflect on the biases we are left with and how we might react with fragility when they are exposed.

**Concurrent Session One**

**The impact of sleep and chronobiology on nociception and chronic pain**

**Session Chair**: Jennifer Daly-Cyr, B. Comm MA, Chronic Pain Network, CircaPain.ca

**Session Abstract**: Sleep is a basic function of most organisms, helping to “reset” physiological clocks. A core set of clock genes regulate circadian (or 24-hour) rhythms, and govern sleep and most other physiological processes. These clock genes also control expression of 10 to 55% of all genes in a tissue- and cell-specific manner. Interestingly, clinical studies have identified rhythmicity in pain outcomes; rodent models also show clear neuro-immune signatures regulated by core clock genes, which are known to impact both acute and chronic pain. However, our understanding of the biopsychosocial impact of circadian rhythms on nociception and pain remain largely unexplored. This session will explore how chronobiology can be used to better understand nociception and pain in Drosophila, rodents, and humans. Findings in the common fruit fly show that painful injury reduces sleep quality and the mechanisms involved differ between inflammatory vs neuropathic injury. In the rodent, we describe the sex-specific regulation of thermal nociception via peripheral neuroimmune mechanisms. Finally, in a cohort of individuals with chronic low back pain, we show that ecological momentary assessment can be used to stratify patients by pain rhythmicity, which correlates with psychosocial outcomes. Our symposium is chaired by a person with lived experience, who will share their personal story of pain rhythms.

**At the end of this presentation, participants will be able to**:

- Recognize the interplay between pain and sleep first evolved ~550 million years ago and may have provided a selective advantage over evolution.
- Evaluate neuroinflammatory mechanisms regulating circadian nociception, and why they may be selective for one sex over the other.
- Identify specific profiles of pain rhythmicity in low back pain and understand how they are associated with psychosocial outcomes.

**Speaker One**

**Neuropathic and inflammatory “pain” reduce sleep quality through distinct mechanisms in the fruit fly**

Greg Neely

The University of Sydney

**Abstract**: Chronic pain patients often experience sleep problems, however the molecular mechanisms involved are largely unknown. To identify conserved genes and pathways governing sleep/pain interactions, we have developed a novel sleep/pain paradigm using the genetically tractable fruit fly. We find painful chronic nerve or inflammatory injury causes reduced sleep quality, and this response can be treated with human painkillers. Mechanistically, nerve injury and inflammatory injury both require peripheral nociceptor function to reduce sleep quality. However, at the level of the CNS, nerve injury and inflammatory pain mechanisms diverge. While nerve injury leads to excitotoxic loss of central inhibition that drives reduced sleep quality, inflammatory injury instead requires glial intermediates, the fly TNF system, and the excitatory amino acid transporter (*EAAT*) expressed on astrocytes. Finally, we show this high throughput system can also help sort through human genetics data and help pinpoint new functional pathways controlling sleep quality in a variety of contexts. These data further support the hypothesis that nociceptive sensitization, where chronic injury leads an animal to enter a heightened state of vigilance, is a core conserved mechanism that protects animals from repeated injury, i.e. that chronic pain was originally an evolutionary adaptation. Overall, the system we describe here can help us better understand the conserved molecular regulation of sleep/pain interactions, and target the underlying pathology driving chronic pain.

**Speaker Two**

**Sex-specific control of thermal nociception is regulated by peripheral neuroimmune mechanisms**

Nader Ghasemlou

Queens University

**Abstract**: Thermal nociception was recently shown to be under circadian control in naïve males, with pain felt as most severe in the middle of the night (rest phase). These results suggested that sleep accounted for ~20% of this rhythmic response, while a stronger endogenous circadian rhythm regulated most of these changes in thermal nociceptive pain (mechanical and cold pain were not assessed). When modeled in the naïve laboratory mouse, we found a similar rhythm in thermal pain outcomes only (assessed using the Hargreaves radiant heat and hot plate tests) and only during the rest phase, while changes in mechanical (von Frey) and cold (acetone) nociception were not affected. Interestingly, these changes in nociception were only evident in male and not female mice. Further investigation of these outcomes identified the peripheral opioid response as critical to this change in nociception following treatment with receptor antagonists. Furthermore, we discovered that peripheral immune cells, including neutrophils, monocytes, and T cells, all display a rhythm in their recruitment into the dorsal root ganglia where sensory neurons reside. Antibody-mediated depletion of these cells was able to eliminate the pain rhythms, as was deletion of the core clock gene Bmal1. Therefore, our work shows that core circadian rhythms are key to pain outcomes, likely by controlling the recruitment of peripheral immune cells to sensory neurons, and that activation of these nociceptors is regulated by an opioid-specific mechanism.

**Speaker Three**

**Profiles of pain rhythmicity in low back pain and associations with psychosocial outcomes**

Gabrielle Pagé

Université de Montréal

**Abstract**: There is increased recognition of the temporal and dynamic nature of pain experiences. Daily maximum pain scores and pain variability have been associated with different outcomes, such as physical and social functioning, fatigue, depression, and general mental health. Recent data from our laboratory shows inter-individual differences in the magnitude and patterns of within-day variations in pain intensity among community individuals living with chronic low back pain, and that such magnitude of pain intensity variability is relatively stable over time. Among individuals with chronic low back pain seen in tertiary care settings, approximately one quarter experience constant mild pain, another quarter experience constant severe pain, one fifth show a rhythmic pattern of pain that increases during the day, and 30% have unclear or mixed pain rhythmicity. Interestingly, it is those individuals with a rhythmic pain pattern who reported reduced levels of depressive symptoms and of opioid consumption compared to individuals with a constant pain pattern. Finally, our research group expanded this work by launching a pan-Canadian study, CircaPain.ca, to investigate pain rhythmicity across chronic pain conditions. To date, around 700 individuals contributed to ecological momentary data and psychosocial outcomes. From this, we understand that distinct pain rhythms are present, including those with daily constant, increasing, or decreasing pain intensity level, and such patterns are associated with pain type, psychological distress and sleep. Better profiling rhythmicity patterns across pain conditions, and understanding how they influence biopsychosocial outcomes can help identify therapeutic treatment targets and facilitate the development of pain management strategies.

**Profiles of pain rhythmicity in low back pain and associations with psychosocial outcomes**

**Session Chair**: Jennifer Tyrrell RN(EC), MN, CPNP-PC, Department of Anesthesia and Pain Medicine, Hospital for Sick Children Lawrence Bloomberg Faculty of Nursing, University of Toronto.

**Session Abstract**: It is known that approximately 20% of youth undergoing major surgery develop chronic post-surgical pain (CPSP) and continue to use opioids months after surgery. It is also known that both chronic pain and long-term opioid use pose a significant burden on children, families, and the health care system in terms of physical, psychological and financial consequences as well as risk of opioid dependency and addiction. This presentation will describe the development of the first pediatric Transitional Pain Service that aims to provide education and support to children and their families throughout their surgical trajectory with the goals of identifying risk factors early, providing timely intervention and preventing the development of CPSP. In this presentation you will hear from patients and families, via video, about their experience with the service and their preferences for education and support. This session will include an in-depth presentation of results from two systematic reviews and meta-analyses on the prevalence and risk factors for pediatric CPSP, what psychosocial factors play a large role in its development and the effectiveness of psychological interventions in preventing CPSP. How this evidence has informed the creation of a novel screening measure for the prediction of pediatric CPSP will be shared. Finally, preliminary results of a randomized control trial evaluating the impact of a CBT based intervention program to target identified psychological risk factors and prevent CPSP in youth will be presented. Preliminary findings will be imparted and the challenges and opportunities in providing timely psychological intervention pre-surgery will be highlighted.

**At the end of this presentation, participants will be able to**:

- Describe the four essential elements of a pediatric Transitional Pain Service and its role in the prevention of chronic post-surgical pain and associated sequelae.
- Identify the risk factors for the development of chronic post-surgical pain in children and youth.
- Describe the role of psychological interventions in the prevention of CPSP.

**Speaker One**

**Using evidence to identify which youth develop chronic post-surgical pain and what can we do about it**

Brittany N. Rosenbloom

The Hospital for Sick Children

**Abstract**: Approximately 20% of youth undergoing major surgery develop chronic post-surgical pain (CPSP) and continue to use opioids months after surgery. Recently, there has been an increase in the number of studies investigating risk factors for the development of CPSP in youth. Dr. Rosenbloom will present results from a recently updated systematic review and meta-analysis on the prevalence and risk factors for pediatric CPSP (n = 2864; registered in PROSPERO). Results indicate that psychosocial factors (e.g., youth anxiety, youth and parent catastrophic thinking about pain) play a large role in the development of CPSP. She will describe evidence-based theories on the complex interplay between these youth and caregiver factors. Using evidence from the meta-analysis, she will discuss methodology that is being used to create a novel screening measure for the prediction of pediatric CPSP. As psychosocial factors are predominant in the prediction of CPSP, psychological interventions are essential for the prevention and treatment of pediatric CPSP. Within the adult literature, psychological interventions (e.g., acceptance and commitment therapy, cognitive-behavioural therapy) delivered to patients undergoing surgery are efficacious in the prevention and treatment of perioperative pain. Dr. Rosenbloom will review the results from a new systematic review and meta-analysis of randomized controlled trials evaluating the effectiveness of psychological interventions (e.g., cognitive-behavioural therapy, distraction) for pediatric perioperative pain (n = 3820). She will discuss the clinical implications of this synthesized body of work.

**Speaker Two**

**Development and implementation of a pediatric transitional pain service**

Lisa Isaac^a,b^

^a^The Hospital for Sick Children Temerty Faculty of Medicine; ^b^University of Toronto Associate Professor, University of Toronto

**Abstract**: Using a biopsychosocial model, Dr. Isaac will discuss the development & implementation of a pediatric Transitional Pain Service (pTPS). The presentation will include a video recording of a discussion with patients and families about their experience with pTPS. This will be contextualized with evidence from the literature around patient preferences regarding discussing their post-acute pain experience and needs with medical specialists. The literature around patient preferences for their healthcare has expanded to include pediatric patients and their caregivers, and specifically as it relates to pain management. As most patients would like more information about expected perioperative trajectories, education and pain management planning around surgery which includes patient preferences, are important components of the transitional pain service. Opioid stewardship is increasingly recognized as a critical component of pain management, given the common and often necessary need for perioperative opioids. The role of the pTPS in pediatric opioid stewardship will be reviewed with evidence from the literature discussing methods of rationalizing opioids and resources while improving pain management and function. Pediatric developmental differences will be highlighted as they pertain to these services, and relative to the adult experience. Dr. Isaac will review the four main elements of an effective pTPS: opioid stewardship, perioperative pain management planning, postoperative and post-acute pain multi-disciplinary specialist treatment and prevention of chronic pain. These four elements support the goal of improved patient experience, improved health, and the potential for decreased healthcare costs.

**Speaker Three**

**A 4 session psychologist delivered pre-surgical program (PPP) for preventing chronic postsurgical pain in youth receiving major surgery - Preliminary data**

Danielle Ruskin^a,b^

^a^Dept of Anesthesia and Pain Medicine and Dept of Psychology Hospital for Sick Children; ^b^Graduate Program in Psychology, York University

**Abstract**: Psychological factors such as children’s anxiety and pain catastrophizing levels along with their confidence in managing pain contribute to the risk of developing chronic post surgical pain (CPSP). Yet, only a small number of studies have been published to describe interventions that target psychological risk factors to prevent CPSP. Dr Ruskin will present preliminary results of a randomized control trial evaluating the impact of a 4 session therapist delivered CBT based intervention program to target psychological risk factors and prevent CPSP in youth. In this trial, 3 sessions of therapist delivered manualized therapy were delivered prior to surgery and the 4th session was delivered 2 weeks post surgery. Additionally, a psychology consultation was conducted with each participant at the outset of treatment to identify specific psychological risk factors. Twenty youth will be randomized to the treatment condition and 20 youth will be randomized to receiving specialty treatment through our hospital’s transitional pain program (i.e., education on pharmacological and non pharmacological strategies but not specific targeted treatment providing non pharmacological strategies). Preliminary findings on our sample (at writing we have N = 24 youth participating) will be presented, including data on feasibility and acceptability of the PPP intervention along with findings on children’s top worries about surgery and psychological consult results. Challenges and opportunities in providing timely psychological intervention pre-surgery will be highlighted.

**Innovations in complex cancer pain research: Learning from real-world clinical experiences to improve research methodology and clinical practice**

**Session Chair**: Maxime Bouchard DCES, Research advocate and person with lived experience.

**Session Co-Chair**: Dr. Lynn Gauthier, Laval University

**Session Abstract**: Pain, among the most common symptoms across the cancer continuum, may be caused by the disease as well as disease-modifying treatments. The presentation of many complex cancer pain syndromes (e.g., post-mastectomy pain, chemotherapy-induced peripheral neuropathy [CIPN], radiation-induced oral mucositis) vary widely within and across syndromes, reflecting different underlying mechanisms, resulting in the need for tailored assessment and treatment. Unfortunately, assessment is often inadequate, and for some cancer pain syndromes there are limited treatments and prevention strategies. In this session, Lynn Gauthier will interview Maxime Bouchard, who lives with complex cancer pain, about the development and progression of this pain through multiple cancer treatments, his experiences of inadequate assessment and management, and the implications of unmanaged pain on treatment decision-making. This conversation will provide real-world context for 3 speakers who will highlight opportunities to improve research methodology to better measure, manage, and develop preventive treatments for complex cancer pain, and the key role of people with lived experience in methodological advancements. Philippe Bérubé-Mercier will present a systematic review of CIPN measure quality and methodological advancements toward the identification of a gold standard measure. Jennifer Gewandter will discuss ACTTION (Analgesic, Anesthetic, Addiction Clinical Trial, Translations, Innovations, Opportunities, and Networks) expert consensus-developed design considerations for CIPN prevention trials. Dale Langford will discuss interindividual differences in the experience of cancer pain, strategies for identifying distinct multidimensional pain profiles, and the value of patients’ perspectives in developing clinically meaningful outcome assessments. An interactive discussion will highlight additional opportunities for real-world experience to inform research methodology.

**At the end of this presentation, participants will be able to**:

- Identify methodological improvements to enhance the quality of ClinROM and PROM in assessing CIPN.
- Develop strategies that can be implemented to optimize the design of CIPN (and other pain) prevention trials.
- Appreciate the tremendous complexity of cancer pain and understand strategies that can be used to better capture the pain experience as a whole.

**Speaker One**

**Improving CIPN measurement by partnering with people with lived experience: Recommendations from a systematic review of the quality of CIPN measures**

Philippe Bérubé-Mercier

Université Laval

**Abstract**: Chemotherapy-induced peripheral neuropathy (CIPN) is a side effect of several neurotoxic agents. Many Patient-Reported Outcome Measures (PROM) and Clinician-Reported Outcome Measures (ClinROM) have been developed to assess CIPN. However, there is no consensus on which measure should be used, limiting assessment and management in real-world settings and contributing to methodological limitations in prevention and treatment trials. In this presentation, Mr. Bérubé-Mercier will review the variability in CIPN conceptualizations contributing to these limitations. Next, he will present a systematic review conducted by a multidisciplinary team including Mr. Bouchard 1) identifying all CIPN PROM and ClinROM for adults receiving/who had previously received neurotoxic chemotherapy for cancer; 2) assessing their quality; and 3) recommending one or more for clinical and research use. MEDLINE, Embase, CINAHL, and PsycINFO were consulted from inception to 12/2021. 93 studies reporting psychometric proprieties of 39 PROM and 32 ClinROM among adults aged ≥18 years, with no language restriction, were included. 14 (35.9%) PROM and 0 ClinROM were developed with input of research participants with CIPN lived experience. No research teams included people with lived experience. Participants received mixed agents (several single agents/combination therapy [62.3%]) or single agents (platinum [9.7%]; taxane [5.4%]). 22.6% of studies included no agent information. Studies included people currently (62.4%) and not currently undergoing chemotherapy (22.6%). 15% of studies included no timing information. COSMIN (COnsensus-based Standards for the selection of health-Measurement Instruments) quality analysis will be reviewed. Methodological improvements will be proposed. Recommendations co-developed with research team members with CIPN lived experience will be discussed.

**Speaker Two**

**Considerations for improving assay sensitivity and clinical relevance of CIPN prevention trials - ACTTION recommendations**

Jennifer Gewandter

University of Rochester

**Abstract**: Chemotherapy-induced peripheral neuropathy (CIPN) occurs in approximately 60% of patients who receive neurotoxic chemotherapies and becomes chronic in approximately 50% of those patients. CIPN symptoms include pain, tingling, cramping, numbness, and weakness. CIPN can cause chemotherapy dose reductions and discontinuation, potentially increasing mortality. CIPN also adversely affects patient quality of life. No preventive or curative treatments for CIPN are available. In order to develop novel therapies that prevent or slow the progression of CIPN, it is imperative to have well-designed clinical trials to increase assay sensitivity (i.e., the ability of the trial to detect a true treatment effect). In this presentation, Dr. Gewandter will discuss clinical trial design considerations for trials of preventive and disease-modifying CIPN treatments that were developed at an expert consensus meeting organized by ACTTION (Analgesic, Anesthetic, Addiction Clinical Trial, Translations, Innovations, Opportunities, and Networks), a U.S. Food and Drug Administration public-partnership. She will focus on considerations for entry criteria, neuropathy assessment, trial endpoints, and feasibility, as well as opportunities to partner with patients with lived experience to optimize trial design. She will also discuss the advantages and limitations of composite endpoints in the context of CIPN trials and more broadly. In general, this discussion will also serve to inform design for RCTs of pain prevention in other areas.

**Speaker Three**

**Complexity of cancer pain and strategies to evaluate patients’ multidimensional pain experiences**

Dale Langford

University of Rochester

**Abstract:** The experience of chronic non-cancer and cancer pain is multidimensional and impacted by a myriad of biopsychosocial factors. An understanding of cancer-related pain is particularly challenging and important given that (1) pain may be due to a number of disease and/or treatment-related factors (surgery, chemotherapy, radiation), (2) pain can negatively impact function, quality of life, and critical treatment decisions (e.g., dose reduction or cessation of critical antineoplastic/anticancer therapies) and (3) pain commonly co-occurs with other deleterious symptoms, such as sleep disturbance, fatigue, and depressive symptoms (known as a “symptom cluster”). Not surprisingly, there is substantial variability in the experience of cancer-related pain. Appreciating that pain does not occur in isolation suggests that a more nuanced and personalized approach to its study and treatment is warranted. To that end, Dr. Langford will highlight individual variability in the experience of pain with some illustrative and interactive examples, discuss biopsychosocial factors associated with the experience of cancer-related pain, outline potential strategies to identify subgroups of individuals with distinct pain (and symptom cluster) profiles, and emphasize the importance of a holistic evaluation of the pain experience. She will also discuss ongoing efforts to develop clinically meaningful global assessment and pain intensity measures that are informed by interviews with people with lived experience of pain. Dr. Langford will propose that efforts to understand the tremendous interindividual variability in the experience of cancer-related pain may provide clinically-relevant risk profiles, reveal novel therapeutic targets for pain prevention and management, and support evidence-based individually-tailored pain management.

**Pain education in canada: New research in pre- and post- licensure learning and outcomes**

**Session Chair**: Iacopo Cioffi DDS PhD, University of Toronto

**Session Abstract**: Pain is recognized as a global health problem with significant impacts on population health and healthcare systems. This problem emerges, in part, from insufficient pain education in both pre- and post-licensure learning contexts. In response, the Canadian Pain Task Force (CPTF) has produced a national pain strategy and action plan to improve awareness, education, and specialized health professional training for pain. This plan includes a call for innovative knowledge translation activities to transform research into practice and reduce disparities in curricula through common learning resources and outcomes. This symposium reports on new Canadian pain educational research across the continuum of pre- to post-licensure learning: impact of an interfaculty pain curriculum for pre-licensure health professions; continuing professional development learning needs of post-licensure health professionals; and opportunities for pain competency outcome measurement across pre- and post-licensure learning contexts. These presentations emphasize CPTF recommendations for reducing disparities in learning opportunities through common learning resources or strategies that are scalable. Presentations emphasize the importance of experiential- and practice-based learning that is patient-centered, collaborative, and comprised of meaningful outcome evaluation. This session will be of interest to health professionals of all backgrounds, educators, policymakers, and researchers interested in pain education and engage attendees with opportunities for informal discussion.

**At the end of this presentation, participants will be able to**:

- Recognize disparities in pain learning opportunities in Canada.
- Articulate the pain learning needs and experiences of Canadian pre-and post-licensure health care professionals.

- Identify opportunities to measure knowledge and competence in pain management.

**Speaker One**

**Pre-licensure health professional student knowledge development in an interfaculty pain curriculum: Lessons learned**

Sylvia Langlois

University of Toronto

**Abstract**: In Canada, 1 in 5 individuals suffers from chronic pain resulting in significant personal and societal costs. Given the growing burden of pain, it is necessary to increase awareness about pain and enhance formal pre-licensure health professional pain education to advance a practice-ready workforce to manage this complex problem. The flagship educational initiative of the University of Toronto Centre for the Study of Pain (UTCSP) is the internationally recognized Interfaculty Pain Curriculum (IPC). The overall aim of the IPC is to prepare pre-licensure health professional students with the knowledge and skills to provide pain care as part of an interprofessional team. In this presentation, we will offer reflections on 10-years of curriculum development with a focus on optimizing student knowledge and beliefs about pain, and their ability to collaboratively develop an interprofessional care plan. Lessons learned include the importance of longitudinal evaluation of knowledge gain and student perspectives on their learning experiences. We will outline the importance of curriculum developers, researchers, and instructors using a framework to better understand the mechanisms behind student learning and competency development and consider opportunities for curriculum innovation.

**Speaker Two**

**Continuing professional development needs in pain management for canadian post-licensure health care professionals**

Craig Dale

University of Toronto

**Abstract**: Continuing Professional Development (CPD) is an important means of improving access to effective patient care. Although pain content has increased significantly in pre-licensure programs, little is known about how post-licensure health professionals advance or maintain competence in pain management. This presentation reports on a cross-sectional self-report web survey investigating Canadian post-licensure health professionals’ CPD needs, activities, and preferred learning modalities for pain management. The survey results demonstrate that most patients encountered in an average week present with pain and learning needs emphasize complex pain conditions. Despite the importance of competency development for complex pain management, recently completed and preferred learning modalities about pain are informal. Presentation recommendations will emphasize how Canadian postlicensure health professionals require greater access to and participation in interactive and multimodal methods of CPD to facilitate competency in evidence-based pain management. This includes the need to create targeted CPD, both formal and informal, to translate best pain evidence into practice. Discussion will include the importance of digital CPD modalities to improve equitable access to learning opportunities.

**Speaker Three**

**Integration of competence-based assessment into pre- and post-licensure pain education**

Samah Hassan

University Health Network

**Abstract**: Pain education has now shifted towards a competency-based education (CBE) approach for both pre-and post-licensure health professionals. CBE is a multifaceted outcomes-based approach that focuses on knowledge, skills, and abilities. The goal of CBE is to ensure that learners achieve the desired patient centered outcomes during training. Pain education has also adopted an interprofessional approach where different professions learn with, from, and about each other. To align with these new educational approaches, we developed and report on an online pain competency-based assessment tool (PCAT) based on the interprofessional pain management core competencies. The PCAT consists of five case scenarios followed by 17 key-feature questions. Pre- and post-licensure health professionals completed the PCAT to assess its psychometric properties through a series of studies. Although the PCAT was primarily developed for post-licensure professionals, evaluations suggest that it is relevant to different pre- and post-licensure health professionals. The PCAT questions demonstrated acceptable reliability (Cronbach’s alpha = 0.7), and substantial stability (intraclass correlation coefficient = 0.85). The PCAT scores significantly distinguished between learners from different levels of competencies. The PCAT scores also showed significant changes in the baseline scores compared to scores post-attending an educational program. The PCAT scores were higher among participants who completed it in groups compared to those who completed it individually reflecting interprofessional competencies. The PCAT offers a first-of-its-kind tool for assessing interprofessional pain core competencies and it sets the foundation for developing profession-specific pain competency assessment tools in the future.

**Concurrent Session Two**

**Sex differences in pain and analgesia mechanisms: Evidence from animal and human brain imaging**

**Session Chair**: Joyce Da Silva PhD, University of Maryland, Baltimore

**Session Abstract**: Females and males differ in brain structure and function. These findings may potentially explain sex differences in pain and efficacy and misuse of certain treatments, such as opioids. In this symposium, brain mechanisms underlying sex differences in distinct pain conditions from both animal and human studies will be presented. Dr. Joyce Da Silva will discuss how connectivity of the periaqueductal gray (PAG) with the whole brain is influenced by sex and age in a healthy state and during osteoarthritis (OA) progression in rats. Using longitudinal fMRI data, alterations in PAG connectivity as OA pain progresses from acute to chronic states will be discussed, as well as a potential mechanism underlying chronic OA pain in elderly patients. Dr. Jose Moron-Concepcion will consider new data reflecting on neural mechanisms underlying sex-specific effects of pain on opioid use. Novel data from an inflammatory pain model in rats will demonstrate how pain alters opioid reward signaling in a sex-specific and time-dependent manner and reveal novel mechanisms underlying opioid misuse liability. Lastly, Dr. Natalie Osborne will discuss fMRI findings from two chronic pain conditions (a form of arthritis called ankylosing spondylitis and carpal tunnel syndrome) that reveal sex-specific patterns of abnormal subgenual anterior cingulate cortex (sgACC) connectivity compared to healthy adults. The influences of age, sex, pain condition and treatment on the sgACC’s role in chronic pain will be discussed. This translational symposium from experimental to clinical settings will help establish meaningful central mechanistic insights into sex differences in pain conditions and opioid use.

**At the end of this presentation, participants will be able to**:

- Reflect on the impact of sex and age on central mechanisms of pain, with focus on functional connectivity of descending pain inhibitory pathways.
- Identify how pain impacts motivational states that may drive drug seeking behavior in a sex-dependent manner.
- Recognize commonalities in basic and clinical studies exhibiting sex differences in brain plasticity in chronic pain.

**Speaker One**

**Using functional MRI to assess sex and age differences in pain processing at the whole-brain level in a preclinical model of chronic pain**

Joyce Da Silva

University of Maryland, Baltimore

**Abstract**: Old age and female sex are risk factors for the development of osteoarthritis (OA) and chronic pain, yet the underlying mechanisms are poorly understood and available treatments are largely ineffective. Increasing evidence suggests that the high OA prevalence in the elderly does not reflect a simple consequence of the biological changes associated with age but rather the complex interplay between age, sex, and pain processing at multiple levels of the neuroaxis. Alterations in descending pain modulatory networks may contribute to the pathophysiological basis for chronic OA pain, and potential therapies could target these networks. This session will present new data on functional connectivity of the periaqueductal gray (PAG), a main core of the descending pain inhibitory pathways, with the whole brain and sex- and age-related changes in a healthy state and during chronic pain progression in rats (early and late phases of OA pain). Data reflecting how sex and age differences in PAG connectivity may affect pain-like behaviors in a preclinical OA model will be presented as well as their clinical applications Moreover, it will be demonstrated that consistently with human studies age significantly impacts OA-like pain making the elderly, particularly females, more vulnerable to chronic pain.

**Speaker Two**

**Pain associated fentanyl intake in males is driven by dynamic activity of VTA dopamine neurons**

Jose Moron-Concepcion

University of Maryland, Baltimore

**Abstract**: Evidence suggests that the proclivity for opioid abuse under pain conditions varies between sexes. Though women are more sensitive to pain, men are more likely to escalate opioid doses, meet diagnostic criteria for opioid use disorder,and die from overdose. Previous reports from our lab indicate that pain disrupts opioid receptor-dependent dopamine (DA) release from the ventral tegmental area (VTA). However, it remains unclear whether pain impacts the ability of VTA DA neurons to respond to opioid reinforcement in a sex-dependent manner. Simultaneous intravenous self-administration and fiber photometry recordings were achieved using wireless in vivo fiber photometry. Every 1-5 days, a wireless, battery-powered transmitter head stage was secured to the optic fiber and GCaMP fluorescence was measured throughout the duration of the session. Pain increased consumption of, and motivation for, fentanyl, selectively in males. These behavioral effects developed over time and were paralleled by sex- and pain-specific effects on tonic VTA DA ΔF/F signals throughout the sessions and phasic VTA DA ΔF/F signals time-locked to fentanyl-reinforced lever presses. Males with pain showed deficits in fentanyl-evoked VTA DA ΔF/F activity at early time points which were amplified over time in a manner that corresponded with increased fentanyl intake. Finally, we found that chemogenetic inhibition of VTA DA neurons during late stages of self-administration was sufficient to normalize fentanyl intake in males with pain and associated VTA DA neuron activity. These findings reveal sexspecific pain-induced adaptations to VTA DA neuron function that underlie maladaptive patterns of opioid use.

**Speaker Three**

**The subgenual anterior cingulate cortex: Sex differences and sex specific abnormalities in chronic pain**

Natalie Osborne

University of Toronto

**Abstract**: Chronic pain is associated with brain reorganization, but despite animal evidence for sex differences in central mechanisms underlying chronic pain development, sex differences in chronic pain-related neuroplasticity in humans are understudied. The subgenual anterior cingulate cortex (sgACC) is important for descending pain modulation, shows sex differences in its functional connectivity (FC) in pain-free individuals and has been proposed as a neurostimulation treatment target for chronic pain. This session focuses on sgACC FC in two chronic pain conditions with sex differences in their prevalence and severity: ankylosing spondylitis (AS, a form of arthritis more common in men) and carpal tunnel syndrome (CTS, a neuropathy more common in women). Resting state fMRI revealed sex differences and sex-specific abnormalities in sgACC FC with sensorimotor, default mode, and salience networks. Overall, abnormal sgACC connectivity was seen in individuals with chronic pain conditions less prevalent in their sex. Specifically, women (but not men) with AS had abnormally high FC with the precuneus and low FC with the dorsolateral prefrontal cortex compared to pain free women. In CTS, sgACC FC was influenced by sex and age, and was associated with abnormally low sgACC FC with the medial prefrontal cortex in men (but not women). A longitudinal analysis revealed pain treatmentrelated plasticity in the sgACC’s FC with regions associated with pain intensity and affect. These findings, along with evidence from graph theory and magnetoencephalography studies, highlight the need to consider sex, age, and chronic pain condition’s influence on sgACC circuitry when developing novel neurostimulation treatments for pain.

**Early life trauma and chronic pain across the lifespan and generations: Identifying mechanisms across species and new intervention targets to break the cycle**

**Session Chair**: Joletta Belton MSc, IASP Global Alliance of Partner’s for Pain Advocacy

**Session Abstract**: Pain and trauma are inextricably linked. Pain is one of the first warning signs of a cascade of mental health issues (PTSD, depression). PTSD and trauma can also precede the development of chronic pain and exacerbate risk, even before children are born. Emerging evidence shows that pain is disproportionately experienced by and undertreated among minoritized and marginalized populations, many of whom experienced higher rates of trauma. In our recent Lancet paper, we argued that living a life with pain can be a catalyst for toxic and traumatic stress given the social/structural barriers that people living with pain face (discrimination, invalidation). To break the intergenerational cycle of trauma and chronic pain, we must examine all levels of analysis (neurobiological, psychological, social, structural), ensure inclusivity and representation of marginalized individuals, generate and implement innovations in prevention and treatment, and ensure that advocacy and policy change are centered. This workshop brings together an international (Canada, US, Australia), cross-disciplinary panel of scientists and an advocate with lived experience of early life trauma and chronic pain who will examine the epidemic of chronic pain and its connection to early life trauma from a bio-psycho-social-structural lens. We will present new emerging experimental, clinical, and preclinical data on the neurobiological, behavioural, and social/interpersonal mechanisms and processes that drive pain across the lifespan and generations. We will also discuss the need for an expanded structural approach to our pain science that promotes equity and inclusivity and targets systemic sources of trauma and toxic stress (racism, misogyny, homophobia, etc.).

**At the end of this presentation, participants will be able to**:

- Define some of the epigenetic mechanisms responsible for the intergenerational transmission of chronic pain risk.
- Recognize new emerging, longitudinal data from both clinical and preclinical models, the key neurobiological and social mechanisms that underlie the trauma-chronic pain relationship in adolescence and its intergenerational transmission.
- Apply validating behaviors to the recovery from early life adversity and the management of pediatric chronic pain.

**Speaker One**

**Utilization of a rodent model of intimate partner violence to understand the influence of in utero trauma on adolescent nociception**

Richelle Mychasiuk

Monash University

**Abstract**: An underlying risk factor for the development of chronic pain is early life trauma which may alter developmental trajectories and result in neuropathological and behavioural phenotypes that predispose individuals to persistent pain. Early life trauma may begin *in utero*. A major and grossly understudied form of trauma *in utero* involves intimate partner violence (IPV) directed towards the mother. IPV predominantly effects women and is a significant form of violence that often escalates during pregnancy. Adolescence in particular is a period of development characterized by functional and physiological neuroplasticity which likely confers evolutionary benefit by allowing them to engage more readily and independently with their environment. However, this sensitivity to environmental factors also corresponds to an increased risk and susceptibility to a plethora of illnesses including chronic pain, depression, and anxiety. While there is no singular known cause from which the transition from acute to chronic pain can be attributed, current literature demonstrates that reorganisation within the mesocorticolimbic system may underpin central sensitisation resulting in pain hypersensitivity. These neuroplastic changes are likely driven by epigenetic regulation. Epigenetic processes occur across the lifespan but are particularly active during prenatal and early postnatal stages. Therefore, we aimed to characterise the epigenetic response that leads to the neuropathological and systemic changes associated with the chronification of pain in adolescents exposed to in utero trauma.

**Speaker Two**

**Disentangling the effects of neurobiology and social factors in the etiology of trauma, pain and its intergenerational transmission**

Melanie Noel

University of Calgary

**Abstract**: Chronic pain is an intergenerational problem transmitted across generations and powerfully influenced by early traumatic and painful experiences. Youth with chronic pain and their parents experience traumatic events and PTSD at high rates and trauma drives chronic pain and disability in a reciprocal fashion. Conceptual models of co-occurring PTSD and chronic pain posit that neurobiological, cognitive behavioral, and social factors drive the chronic pain-PTSD relationship across generations; however, there is a paucity of prospective research during adolescence, the period during which chronic pain often first emerges. We have new emerging, translational data from both clinical and preclinical models that provide compelling evidence of key neurobiological and social mechanisms that underlie the relationship between early life trauma/adversity and chronic pain in adolescence and its intergenerational transmission. Dr. Noel will present new data from two clinical studies (a birth cohort [n=3000] and clinical cohort [N=150] of parent-child dyads) examining whether early trauma of parents and youth influence the later development of chronic pain, chronic pain predicts first onset of later suicidality and depression, and these relationships are either buffered or exacerbated by parental behaviors. This complements international collaborative work examining two preclinical rodent models, demonstrating how early life stress before and at birth leads to pain problems in adolescence through key epigenetic, inflammatory, and microbiome changes. A new, trauma-informed, transdiagnostic, family program that Dr. Noel developed to prevent pain and mental health issues in youth at high risk for these issues (i.e., children of parents experiencing trauma and chronic pain) will be discussed.

**Speaker Three**

**Validation as a viable intervention target for promoting recovery from early life adversity and managing chronic pain**

Chad Shenk

Penn State University

**Abstract**: Early life adversity is a well-established risk factor for a variety of adverse health conditions in childhood and adulthood, notably chronic pain. This raises the opportunity for identifying additional environmental factors that could serve as intervention targets to prevent or mitigate these long-term health risks. Validation is a dynamic, lag-sequential process where one person, such as a caregiver or medical provider, communicates acceptance, understanding, and valuing of another person’s subjective experience. Validation has been proposed as a transdiagnostic factor for promoting resilience in the onset and course of a variety of health conditions because it in turn targets the thoughts, emotions, memories, and physiology that make living with early life adversity and managing chronic pain more difficult. This presentation will accomplish three goals: 1) review the evidence base supporting validation as a viable intervention target using results from experimental, observational, and clinical research, 2) present results from a prospective cohort study (N=439 dyads; Child age: 8-13 years) directly observing and independently rating live patterns of validation as a source of resilience in preventing adverse pediatric health following exposure to child maltreatment, and 3) outline the rationale and methods for targeting validation in the management of pediatric chronic pain, including opportunities for caregiver-child and patient-provider interactions. Validation is a specific intervention target, with decades of empirical support, that has considerable potential to mitigate the risk for and improve the management of pediatric chronic pain.

**Classification of chronic primary pain in ICD 11 - does nociplastic pain terminology allow for better diagnostic categorization and enhanced treatment opportunities?**

**Session Chair**: Nimish Mittal MBBD MD MSc, University of Toronto.

**Session Abstract**: Chronic primary pain is a new classification adopted by the ICD 11 for pain conditions that do not belong to the typical nociceptive or neuropathic domains and are accompanied by significant emotional distress or functional disability. Nociplastic pain is a new mechanistic term for chronic primary pain conditions devoid of observable tissue damage or abnormality. Classification of chronic primary pain is a step forward in embracing the biopsychosocial concept of chronic pain while moving away from the dichotomy of physical versus psychological pain and avoiding ambiguity of pain labels such as functional, non-specific, somatoform, etc. Moreover, the ICD 11 classification of chronic primary pain provides the foundation for a paradigm shift toward comprehensive multimodal centrally directed treatment strategies.

This panel discussion will introduce participants to the classification structure of ICD 11 chronic primary pain and its subcategories. The panel will discuss common pain conditions, previously ambiguously labelled, in accordance with the new framework. Early recognition and appropriate comprehensive management strategies will optimize patient care. To promote debate, the panel will also discuss opportunities for expanding the definition of nociplastic pain. Pain physicians with extensive knowledge of chronic pain conditions and the four-decade evolution of chronic pain classification are among the presenters.

Each speaker will give a 15-minute presentation, followed by a 30-minute panel debate on opportunities and pitfalls in the ICD 11 classification of chronic primary pain versus non-primary pain. The final fifteen minutes will be devoted to audience questions and robust discussion of this new classification framework’s clinical implications.

**At the end of this presentation, participants will be able to**:

- Describe the new framework and classification of chronic primary pain according to the ICD 11 chronic pain classification.
- Review the new structure of chronic primary musculoskeletal pains and discuss chronic primary widespread pain and common masked pain conditions that require differential diagnosis.
- Describe the limitations of the current framework of nociplastic pain in relation to the classification of chronic primary pain.

**Speaker One**

**Should we look to nociplastic pains as the holy grail for chronic primary pains diagnosis in ICD 11?**

Nimish Mittal

University of Toronto

**Abstract**: Chronic Primary Pain (CPP) is a new diagnostic category in ICD 11 pain classification created to better recognize the biopsychological model of pain rather than the mutually exclusive dichotomy of physical vs psychological underpinnings and to avoid invalidating and undermining terms like “nonspecific” and “functional.” The basis of CPP is the newly coined mechanistic term nociplastic pains, which is used to describe poorly understood pain mechanisms that influence functional changes in the nociceptive pathways. This term is used to describe pain mechanisms that are not fully understood and that affect the functional changes in the nociceptive pathways. However, the new classification and the proposed change of terminology can be confusing for both clinicians and researchers. This is because nociplastic pain does not have any diagnostic tests that are clinically useful and reliable, and it is currently graded using a certainty model as either possible or probable, but not certain. During this session, the new criteria for chronic primary pain in the context of nociplastic pains according to ICD 11 will be discussed. Additionally, the nociplastic diagnostic algorithm and grading system will be illustrated with the help of case vignettes. It will be discussed how to interpret clinical findings in order to determine the pain phenotype in mechanistic terms, eliminate the possibility of a differential diagnosis, and determine the relevance of these findings in order to pursue effective treatment strategies.

**Speaker Two**

**Can regional fibromyalgia be an explanation for some baffling pain conditions?**

Mary-Ann Fitzcharles

McGill University

**Abstract**: Is there such a thing as regional fibromyalgia syndrome? How can we address a pain condition that is confined to a localized region (eg. a 20 cm diameter somewhat circular area on the posterior chest wall), without any anatomical explanation? Can the newly proposed subcategory of chronic primary pain, namely chronic primary musculoskeletal (MSK) pain, be an explanation? The neurophysiologic underpinnings of chronic primary pain conditions is believed to be nervous system sensitization, with nociplastic pain proposed as a terminology to describe this new third mechanism of pain. With ample study of fibromyalgia as an example of nociplastic pain, it is possible that some regional pains that have been difficult to understand or categorize may now be better understood. This session will examine the proposed clinical criteria for a diagnosis of chronic primary MSK pain, discuss challenges in differentiating it from myofascial pain syndrome, and stimulate thought about uptake in both the patient and medical community. The value of this session will be for healthcare professionals to apply this new understanding to care of patients with pain conditions that have been difficult to understand.

**Speaker Three**

**Is there a “home” for patients characterized as “unclassified” according to the proposed ICD 11 clinical criteria and grading system for the diagnosis of nociplastic pain?**

Angela Mailis

University of Toronto

**Abstract**: There is no question that the introduction of the mechanistic term “nociplastic pain” has created a vivid and ongoing debate. This talk will concentrate on the revised flowchart illustrating the clinical criteria and grading system for the diagnosis of nociplastic pain conditions, paying particular attention to patients who have non-neuropathic, non-nociceptive and non-nociplastic pain (as they lack the mandatory hypersensitivity criterion in multiple testing modalities). This group of patients are orphans, in “no-man’s land,” labelled in the flowchart as “not classifiable.” Based on a recent QST study, almost a third of patients with non-neuropathic/ non-nociceptive regional or widespread pain, have sensory loss (hyposensitivity), instead of gain (hypersensitivity) to cold, heat, and mechanical stimuli. These patients meet all but one criterion for nociplastic pain, as they experience regional/non discreet or widespread pain >3 months, in the absence of structural changes, as well as sleep issues, fatigue, CNS comorbidities and mood/anxiety disorders.

Clinically, such patients may present with sensory deficits to all cutaneous and deep modalities such as heat, cold, touch, deep pressure and even vibration sense (the lateral conducted through the posterior columns, not the spinothalamic system), or may display gain in some modalities and deficit in others. The latter subgroup of mixed gains/deficits, thus, is serving as an intermediate, between the current nociplastic framework and those unclassifiable patients with solely sensory deficits (loss). The speaker will present case examples, review neuroimaging evidence of altered central processing, speculate on mechanisms of enhanced inhibition/ disturbed conditioned pain modulation, and question whether the term nociplastic could be expanded to recognize this sizeable subcategory of “orphan” patients, proposing that centrally mediated gains and deficits are two sides of the same coin, representing centrally mediated neuroplastic changes.

**CIHR hacks: Strategies for getting trainee funding**

**Session Chair**: Dr. Rebecca Pillai Riddell PhD, CPsych, FCAHS, York University

**Session Abstract**: The Canadian Institutes of Health Research provide generous scholarships for Masters, Doctoral, and Postdoctoral level trainees. CIHR Trainee applications require a significant time investment to prepare the different pieces. Is your application package competitive? How do you write a convincing Research Training Environment section? Who are the ideal referees? Join Dr. Rebecca Pillai Riddell to learn about answers to these questions and her strategies for before, during, and after the application process. After the mentorship lecture, there will be an ‘Ask Me Anything’ opportunity for trainees to chat with Pain research mentors about topics relevant to their career development.

**At the end of this presentation, participants will be able to**:

- Conducting a training session to improve the trainees and increase trainees’ proficiency on the obtention of funding through the Canadian Institutes of Health Research (CIHR) imparted by Dr. Rebecca Pillai.
- Fostering collaborative relationships and knowledge exchange between research trainees from all stages and well-settled professionals all with a shared focus on pain.
- Providing the CPS trainees, a safe space to understand how intersectional identities and life experiences mold the career of established professionals in the pain world.

**Concurrent Session Three**

**Contribution of non-neuronal cells to sex differences in nociception and the transition from acute to chronic pain**

**Session Chair**: Giannina Descalzi PhD, University of Guelph

**Session Abstract**: While much attention has been paid to neuronal responses to injury and disease in acute and chronic pain, relatively little is known regarding the contribution of non-neuronal cells. Circulating and skin-infiltrating peripheral immune cells, including those of the myeloid (e.g., neutrophils and monocytes) and lymphoid lineage (e.g. T and B cells), respond to pain peripherally and can also be recruited into the nervous system. Meanwhile, glial cells in the spinal cord and brain (e.g., astrocytes and microglia) are the first line of defense to CNS injury, where they play an immunomodulatory role. Mounting evidence indicates that these peripheral and central neuro-immune interactions are not only critical for nociception and the development/maintenance of chronic pain, but that they show significant sexual dimorphism, and thus may mediate the observed sex differences of pain. The purpose of this symposium is to bring together researchers studying the contribution of sex to the neuroinflammatory response in central and peripheral pain-related mechanisms.

This symposium will feature three speakers, followed by a panel discussion. Presentations will highlight recent investigations into the sexually dimorphic response of immune cells and their impact on the nervous system, including 1) the impact of Ly6C+ monocytes on the resolution of inflammatory pain (Dr. Geoffroy Laumet, Michigan State University); 2) microglial heterogeneity in human and mouse spinal cord in the transition from acute to chronic pain (Dr. Vivianne Tawfik, Stanford University); and 3) astrocyte-neuronal lactate shuttling in the cingulate cortex of mice with chronic inflammatory pain (Dr. Giannina Descalzi, Guelph University).

**At the end of this presentation, participants will be able to**:

- Recognize the involvement of peripheral immune cells in the development and resolution of chronic pain.
- Describe microglia activation in the spinal cord, and how they contribute to chronic pain in a sexually dimorphic manner.
- Describe how chronic inflammatory pain engages astrocyte-neuronal metabolic coupling in the cingulate cortex of mice in a sexually dimorphic manner.

**Speaker One**

**Peripheral IL-10-producing monocytes contribute to sex difference of pain resolution in mice and humans**

Geoffroy Laumet

Michigan State University

**Abstract**: Epidemiological studies showed that the prevalence of chronic pain is 20% higher in women than in men. Therefore, to reduce the inequality of pain management, it is important to understand the mechanisms underlying this sex difference. Emerging data indicate that neuroimmune interactions contribute to sex differences in the development of chronic pain. But whether neuroimmune interactions contribute to sexual dimorphism in the resolution of pain remains elusive. Additionally, while the skin is often the site of injury, cutaneous neuroimmune interactions have been underinvestigated in animal models of pain. Here, in a model of inflammatory pain, cutaneous immunophenotyping indicate sexual dimorphism in immune cell infiltration in the inflamed paw. Strikingly, there is a more important infiltration of Ly6C+ monocytes and interleukin (IL)-10 upregulation in male mice compared to females and this higher level of IL-10-producing monocytes in males is associated with a faster resolution of pain hypersensitivity. Manipulation of hormonal levels by either ovariectomy, orchidectomy, or addition of testosterone switches the levels of IL-10-producing monocytes similar to the other sex. Excitingly, similar data were observed in a large cohort of human patients; following car accident, males have higher levels of IL-10 and monocytes and faster resolution of pain. The levels of IL-10 and monocytes correlate with the speed of recovery in male patients. Our data, from mice and humans, point out to a critical role of IL-10-producing monocytes for the sexual dimorphism of pain resolution.

**Speaker Two**

**Pro-resolution microglia abrogate the transition from acute-to-chronic pain in a sex-independent manner**

Vivianne L. Tawfik

Stanford University

**Abstract**: Activated myeloid-lineage cells, macrophages peripherally and microglia centrally, contribute to the acute-to-chronic pain transition in a sexually dimorphic manner, however, little is known about whether and how microglia may also serve pro-resolution functions. We took advantage of Cx3CR1-CreERT2- eYFP;iDTRlox-STOP-lox microglia cKO mouse to investigate the phenotype of microglia that repopulate in the spinal cord after depletion in the context of peripheral injury. We first performed this microglial depletion at multiple time points after peripheral injury to see if there was a behavioral effect of modulating microglia. We saw a striking decrease in mechanical allodynia in males and females when depletion was performed at 3, but not 7, weeks after injury. Importantly, we found that after depletion microglia repopulate over the course of 14 days almost to baseline complexity. Since allodynia remained low despite repopulated microglia, we hypothesized that they may represent a pro-resolution phenotype. We then further explored the heterogeneity of these cells using sorted cell RNA sequencing. Using a new dataset generated on human spinal microglia using single nuclei RNA sequencing we then cross-referenced our mouse findings to identify the most relevant microglial genes. Overall, we conclude that microglia may serve sex-independent pro-resolution functions after peripheral injury and present several microglia gene targets for further exploration in various pain conditions.

**Speaker Three**

**Astrocyte-neuronal iactate shuttling in the mouse cingulate cortex drives chronic pain development**

Giannina Descalzi

University of Guelph

**Abstract**: Human and rodent neuroimaging studies indicate that chronic pain corresponds with reorganization of an emotion-pain brain circuit, and evidence indicates that neuroplasticity of the anterior cingulate cortex (ACC) is a critical step in this reorganization. We previously showed chronic pain and fear learning enhance neuronal excitability and induce similar plasticity-related gene expression changes in the anterior cingulate cortex of mice. We recently showed that fear learning requires astrocyteneuronal lactate shuttling (ANLS) in the dorsal hippocampus, and that ANLS is necessary for learninginduced associated molecular changes, including increases in plasticity-related gene expression. Here we present data that indicate that ANLS in the ACC may also be involved in neuroplasticity associated with murine models of chronic pain. Recent findings from our lab indicate that astrocyte-neuronal lactate shuttling is involved in acute and persistent stages of inflammatory pain, in a sex specific manner. Whereas both male and female mice show rapid increases in lactate levels in the ACC during the early stages of inflammatory pain, only male mice, show a sustained increase in lactate levels after 7 days of inflammatory pain. Accordingly, chronic inflammatory pain alters proteins involved in lactate metabolism and shuttling in a sexually dimorphic manner. Notably, disrupting astrocyte-neuronal lactate shuttling in the ACC reduces persistent inflammatory pain, but does not affect acute nociceptive thresholds. Our data indicates that there are sex differences in astrocyte-neuronal coupling during chronic pain development.

**The burden of not knowing: How diagnostic and sensory uncertainty impact on pain outcomes and chronic pain disability**

**Session Chair**: Ann Meulders PhD, Maastricht University

**Session Abstract**: Learning to predict danger is adaptive; it assists in anticipating and avoiding harm. Pain is a salient, alarming experience making it a potent motivator for learning. We learn to detect stimuli predicting the occurrence of pain and alter our behavior accordingly to minimise their impact. Such protective responses (fear/avoidance) are adaptive and promote recovery, but when pain no longer signals bodily harm, they may derail into chronic pain. Many people with chronic pain experience uncertainty about pain and its causes, as well as uncertainty regarding their own movement/body location, which may hamper predictive learning, leading to sustained anxiety, fostering overprotective behavior, and increasing fear, avoidance and disability.

This symposium brings together cutting-edge research on how diagnostic and sensory uncertainty can affect fear/avoidance learning processes, contribute to pain persistence, and pain-related disability and how these processes can be targeted in treatment. First, A/Prof. Meulders will present new data using an operant avoidance learning paradigm showing that people with poor proprioceptive accuracy (i.e., uncertain about their own movements), show overprotective behavior. Second, A/Prof Stanton will present longitudinal data from older adults with osteoarthritis undergoing total knee replacement, showing that early post-surgical perceptual dysfunction (uncertainty about their own movement/body location) only occurs in those who develop persistent post-surgical pain. Finally, Dr. Neville will present novel data examining diagnostic uncertainty in youth with chronic pain and their parents, demonstrating its dynamic character, i.e., parent’s shifts from uncertain to certain about their child’s diagnosis is linked to improvement in youth pain disability over time.

**At the end of this presentation, participants will be able to**:

- Define the diagnostic uncertainty in the context of pediatric chronic pain and how changes in parental uncertainty are connected to children’s pain outcomes.
- Explore the role of sensory uncertainty in pain responses and protective behavior.
- Evaluate how diagnostic and sensory uncertainty can be targeted therapeutically.

**Speaker One**

**Being uncertain about your own movement: Poor proprioceptive accuracy is associated with overprotective behavior**

Ann Meulders

Maastricht University

**Abstract**: Learning to predict bodily threat enables us to initiate defensive responses including increased arousal, self-reported fear, but also recuperative avoidance and safety-seeking behaviors. These protective behaviors are adaptive in the acute pain stage, but when they persist after normal healing time or generalize to safe movements and situations, they may foster disability. The imprecision hypothesis of chronic pain proposes that when stimuli associated with pain are poorly encoded, stimulus generalization may lead to excessive spreading of pain. According to this hypothesis, proprioceptive accuracy may be an important contributor to motor learning and memory. Interestingly, proprioceptive accuracy is impaired in various chronic pain conditions, prompting the question whether being uncertain about your own movement is related to avoidance becoming excessive. Yet to date, research investigating the relationship between proprioceptive accuracy and overprotective behavior is virtually non-existent. I will present new data investigating this hypothesis in an operant conditioning paradigm in which healthy participants could learn to perform robotic arm-reaching movements to avoid a pain stimulus. We used a new dynamic movement reproduction task to assess proprioceptive accuracy. Results supported our hypothesis that participants with poorer accuracy show excessive avoidance. Furthermore, exploratory analyses indicated that proprioceptive accuracy was reduced after conditioning. Interestingly, worsened proprioceptive accuracy was associated with overprotective behavior and higher trait fear of pain scores. This study is the first to highlight the role of proprioceptive accuracy in avoidance of pain-associated movements, and points toward the potential of training accuracy to tackle chronic pain disability.

**Speaker Two**

**Sensory uncertainty about knee movement and location matters: Perceptual dysfunction is associated with the development of persistent post-surgical pain after joint replacement**

Tasha Stanton

University of South Australia

**Abstract**: Knee osteoarthritis (KOA) is a leading cause of disability in older adults, with total knee replacement (TKR) surgery considered when pain and functional impairment are severe. Unfortunately, up to 34% of people have suboptimal long-term pain outcomes post-TKR, and in 15% this pain is moderate-severe. It is difficult to predict who will develop persistent post-surgical pain (PPSP). Recent work has demonstrated that people with KOA experience sensory uncertainty (e.g., perceptual dysfunction) relating to their own knee – they find it difficult to localise and/or move their knee/lower leg – with greater sensory uncertainty associated with higher pain and greater disability. In addition, people with KOA who have lower levels of perceptual dysfunction experience more benefit from exercise. These findings raise the possibility that this novel factor – sensory uncertainty – may play a role in PPSP following TKR, possibly by contributing to pain directly or indirectly via maladaptive protective responses (as would be predicted by theories of predictive processing and associative learning, respectively) or by impairing engagement/outcomes with post-surgical rehabilitation. New data from a longitudinal inception cohort of people with KOA undergoing TKR shows that PPSP was common, affecting 53% of participants at 6 months and 34% at 12 months, but unpredictable from baseline patient characteristics. However, perceptual dysfunction, occurring as early at 1-month post-surgery, was present in those who developed PPSP, but not in those who recovered. These findings suggest that sensory uncertainty may play a role in pain persistence following TKR surgery and that therapeutic interventions targeting sensory uncertainty may prove beneficial.

**Speaker Three**

**Understanding diagnostic uncertainty over time and across the pediatric chronic pain care journey**

Alex Neville

Stanford School of Medicine

**Abstract**: Over one third of youth in tertiary level care for chronic primary pain and their parents experience diagnostic uncertainty (DU), which is linked to worse youth pain outcomes. DU has been shown to be a relational and evolving process, which unfolds within the clinical encounters youth and parents experience along their pain journey. While mounting evidence demonstrates that DU is a debilitating phenomenon that can impede recovery, research has yet to explore how DU may change over time and within tertiary level pediatric chronic pain care. Building on previous research demonstrating the prevalence and impact of DU, Dr. Alexandra Neville will discuss the role of the clinical encounter in influencing uncertainty about a diagnosis of chronic primary pain and present new empirical data investigating DU across the tertiary level pain care journey. She will discuss how differing explanations for pain, validation and invalidation from clinicians, and messages of certainty and uncertainty about the cause of pain influences youth’s and parents’ experiences of diagnostic uncertainty. She will share findings from in-depth qualitative interviews characterizing the experiences of DU among youth with chronic musculoskeletal pain and their parents across their pain care journeys. Lastly, using international survey data, she will discuss how DU is dynamic and can change over time. Further, changes in parent DU from uncertain at baseline to certain 12-months later are linked to improved youth pain disability.

**Mechanisms of non-pharmacological and complementary analgesic methods: Evidence from brain imaging**

**Session Chair**: M. Catherine Bushnell, Harold Griffith Professor Emerita, McGill University Scientist Emerita, National Institutes of Health President, International Association for the Study of Pain.

**Session Abstract**: Chronic pain affects up to 20% of the Canadian population, and significantly reduces the quality of life. Unfortunately, pharmacological treatment options for pain are often ineffective in reducing pain, and are associated with significant side effects. Nerve blocks are effective, but require clinical expertise and can be associated with significant complications. Specialized multimodal pain management clinics in tertiary and quaternary centres are often very expensive, labour intensive, and have very long waitlists. As a result, patients with chronic pain are not receiving adequate care, and there is a clear need for new, innovative therapeutic approaches.

This symposium, chaired by Dr. M. Catherine Bushnell, will begin by describing the need for complementary, non-invasive treatment modalities, and their impact on patient’s quality of life, and potential to reduce the impact of chronic pain. Next, Dr. Fadel Zeidan will discuss the neural, psychological, and physiological mechanisms supporting the self-regulation of pain using mindfulness meditation. Next, Dr. Yang Wang will present her work on the impact of sleep on placebo analgesia in patients with chronic pain. Finally, Dr. Massieh Moayedi will present novel findings on the efficacy of peripheral magnetic stimulation (PMS) as non-pharmacological treatment modality that leads to non-invasive analgesia through peripheral neuromodulation.

Significance: The symposium will discuss the latest cutting edge research in non-invasive, non-pharmacological pain modulatory approaches, and aims to increase awareness of these modalities and their neural mechanisms. This will lay the groundwork for future work to develop novel, affordable and effective therapeutic options.

**At the end of this presentation, participants will be able to**:

- Evaluate the efficacy of different non-pharmacological analgesic approaches.
- Assess differences and overlap in the mechanisms of different non-pharmacological analgesic approaches.
- Develop an understanding of factors that can affect analgesic mechanisms.

**Speaker One**

**Gain sleep to gain placebo effects: Poor sleep quality impairs magnitude and likelihood to respond to placebos**

Yang Wang

University of Maryland, Baltimore

**Abstract:** Pharmacological knockout of REM sleep contributes to improve placebo effects in healthy participants in laboratory settings. It is unknown whether similar effects persist in those people who suffer from sleep disturbance and disorders such chronic pain. To address this gap in literature, we investigated the influence of sleep disturbance on placebo effects.

In a cohort of 277 chronic pain (CP) and 189 healthy control (HC) participants, we measured sleep patterns via the Pittsburg Sleep Quality Index (PSQI) and Insomnia Severity Index (ISI) assessed over the past month before testing experimental placebo effects. Placebo effects were elicited using thermal heat stimulations delivered with visual cues in accordance with a classical conditioning and verbal suggestion paradigm. Placebo ratings were assessed using a visual analogue scale ranging from 0 = no pain at all to 100 = maximum tolerable pain.

We observed a higher prevalence of clinical insomnia and poor sleep quality in CP participants (χ2 = 40.33, p < 0.001). In CP, those characterized by poor sleep quality and presence of insomnia exhibited significantly smaller placebo effects compared to those who had good sleep quality and no insomnia (p < 0.001). In both CP and HC participants, restricted sleep duration (< 6 hours per night) was associated with reduced magnitude of placebo effects (F2,2740 = 3.22, p = 0.040). To our knowledge, this is the first study shedding light on the relationship between sleep disturbances and placebo effects suggesting that long-term poor sleep quality could impair the capability to activate endogenous modulatory systems (i.e., placebo effects) and therefore, the likelihood to respond to placebos.

**Speaker Two**

**Mindfulness meditation-induced chronic low back pain relief is driven by unique mechanisms**

Fadel Zeidan

University of California, San Diego

**Abstract**: Dr. Zeidan will present new findings demonstrating that mindfulness meditation engages unique endogenous and neural mechanisms to attenuate pain. A double-blinded, placebo-controlled crossover designed clinical trial examined if mindfulness meditation (n =30) could produce pain reductions in response to an acutely exacerbated (via straight leg raise test) as compared to a placebo-mindfulness (n = 29) condition during opioidergic signaling blockage and placebo-saline. After administering the straight leg raise test, mindfulness meditation significantly reduced pain unpleasantness by 34% and pain intensity ratings by 44% during saline infusion. Study hypotheses were confirmed. Mindfulness significantly reduced low back pain intensity and unpleasantness ratings during IV opioidergic antagonism. In contrast, non-mindfulness meditation, a technique practiced by taking deep and slow breaths, significantly lowered movement-evoked chronic low back pain intensity and unpleasantness ratings during IV saline but failed to significantly reduce back pain during naloxone infusion. In another study, 40 pain-free participants were randomized to four, 20-minute sessions of mindfulness training or book listening. After the interventions, subjects rested and then meditated (mindfulness) or continued to rest (controls) during noxious heat (49°C) and fMRI acquisition. Visual analog scale pain ratings were collected. Mindfulness reduced pain when compared to controls. Mindfulness-based analgesia was moderated by weaker thalamic-precuneal connectivity and ventromedial PFC deactivation, respectively. These data indicate that mindfulness meditation weakens cortical self-referential midline processing of ascending nociceptive inputs to promote a sensory-egocentric decoupling mechanism. Together, these studies demonstrate that mindfulness meditation directly modifies nociception and corresponding pain through multiple, novel mechanisms.

**Speaker Three**

**Repetitive magnetic stimulation for pain modulation**

Massieh Moayedi

University of Toronto

**Abstract**: Pain poses the largest health related burden on society. Despite its prevalence, there are few effective pain management tools with few side effects—especially for chronic pain. One frequently used neuromodulation device is the non-invasive Transcutaneous Electrical Nerve Stimulation (TENS), which is an effective analgesic for a number of neuropathic pain disorders. However, given that TENS uses surface electrode to deliver electrical impulses, it has limited depth and causes discomfort. Peripheral magnetic stimulation (PMS) is a novel non-pharmacological treatment modality that can potentially lead to non-invasive analgesia through neuromodulation. While there is some evidence that repeated PMS (rPMS) can reduce pain, to date there are no high quality controlled studies that demonstrate its efficacy. Hence, there is a clear need to compare the efficacy of existing rPMS treatment paradigms for pain reduction.

First, Dr. Moayedi will present a case study demonstrating the use and efficacy of rPMS in a patient with refractory chronic pain due to glossopharyngeal neuralgia. Next, he will present novel data comparing rPMS and TENS on pain intensity, pain unpleasantness and secondary hyperalgesia to two experimental heat pain models. Further, he will present the effect of rPMS on an objective biomarker of pain sensitivity—peak alpha frequency, as measured by electroencephalography. Together, these data present the promise of a novel, non-invasive and non-pharmacological treatment modality for pain.

**Addressing the gap: Innovative pediatric pain program solutions to address the growing demand**

**Session Chair**: Nivez Rasic MD FRCPC (Anesthesiology and Pain Medicine), Clinical Associate Professor, University of Calgary

**Session Abstract**: Chronic pain is an epidemic that impacts 1 in 5 children; negatively affecting their physical, emotional and social health, with deleterious consequences extending into adulthood. We know that early and effective treatment of pain leads to improved outcomes for children and youth with chronic pain, yet major barriers exist to access effective, comprehensive care. Children with pain currently have inconsistent, insufficient and delayed access to pain services, along with a scarcity of trained health professionals to address the overwhelming need. Waitlists for children to receive pain care are lengthy, made worse by the Covid-19 pandemic, allowing chronic pain to become entrenched and more challenging to treat. Innovative solutions to address this growing demand are urgent and imperative. A youth PWLE will co-chair this symposium with Dr. Rasic and weave in their experience, journey and expertise with chronic pain as they sought treatment. Three unique perspectives offering innovative pain management program solutions will be explored: 1) approach to design and implementation of pediatric pain programs in a high resource environment by Nurse Specialist Laura Rayner (Alberta Health Services, Calgary, AB); 2) feasibility of implementing a new interdisciplinary pediatric pain services in a rural, underserviced area (Alaska) by Dr. Wendy Gaultney (Neuroversion, Anchorage, Alaska); and 3) scalable technologic solutions to prevent the development of chronic pain with Dr. Jennifer Rabbitts (University of Washington, Seattle, WA). Together, we will explore, discuss and debate novel approaches to addressing the epidemic of chronic pain and seek solutions through a moderated symposium and panel discussion format.

**At the end of this presentation, participants will be able to**:

- Identify and discuss key elements of successful project implementation from both an art and science perspective for pediatric pain programs within tertiary care centers.
- Describe relevant processes of pediatric pain program development and expansion in remote, underserviced regions.
- Discuss development and feasibility results of a scalable peri-operative digital health intervention teaching cognitive-behavioral skills to prevent chronic pain in adolescents undergoing spinal fusion surgery.

**Speaker One**

**Balancing the art and science of clinical change: Real world strategies and lessons to plan, develop and sustain pain programs**

Laura Rayner

The Vi Riddell Children’s Pain & Rehabilitation Centre, Alberta Health Services

**Abstract**: Implementing and sustaining pain programs and supports within tertiary care centres can be challenging. It requires implementation science and art to create effective and purposeful change in the dynamic, stressful, and financially constrained healthcare system. The Alberta Children’s Hospital has been successful in initiating and maintaining a variety of supports and programs to address the gaps in pain care across the spectrum. These include: the first multi-week Intensive Pain Rehabilitation Program (IPRP) in Canada, the site/province wide Commitment to Comfort, and the newly implemented Extended Pain Service, impacting thousands of patients and families. These programs are in different stages of development and have varying spread and scale impact. Needs assessments, benchmarking, and logic models were created to support and plan the programs. With the support of leadership, multidisciplinary pain subcommittees were created to facilitate clinical implementation. In collaboration with researchers, evaluation and extensive outcome measures are completed through REDCap and Alberta Health Services survey platforms, informing many of the ongoing changes and supporting clinical work and patient progress. Throughout the process of developing these programs, a few key elements have been consistent throughout: supportive leadership, clinical and patient expertise, planning, coordination, leveraging health systems and evaluation. Each development and change offered new lessons in how to be an adaptive communicator, develop relationships, and complete projects with energy and humor. These aspects will be identified and discussed, as well as the key learnings and challenges that could be applicable to any pain project or program development.

**Speaker Two**

**Developing and expanding pediatric pain services in Alaska**

Wendy Gaultney

Neuroversion

**Abstract**: Alaska is the largest US state and has the lowest population density. There are approximately 180,000 children and adolescents in Alaska. According to global estimates, approximately 45,000 will meet IASP criteria for chronic pain at some time during childhood/adolescence. Many of these youth live in rural communities, far from major healthcare centers. Limited access to healthcare is especially notable for Alaska Natives, who make up 15.7% of the population and are a marginalized and vulnerable group. To address the largely unknown and unmet need for high quality pediatric pain care in Alaska, we formed an interdisciplinary program in Anchorage, Alaska in May 2021. Our team consists of a physician, psychologist, a medical assistant and a physical therapist. During our first year, we focused on processes related to offering outpatient interdisciplinary pediatric pain services in a privately owned pain clinic. Additionally, we started collecting data to evaluate outcomes. We will discuss successes and challenges associated with early program development. During our second year we have dedicated efforts to expanding access beyond the outpatient clinic walls to prioritize equitable access to pediatric pain resources. Activities have included collaborations within the school systems, existing healthcare and academic systems, and growing our social media presence. We will share highlights and lessons learned during this ongoing phase of program expansion.

**Speaker Three**

**Scalable solutions to prevention of chronic pediatric pain**

Jennifer Rabbitts

Associate Professor of Anaesthesiology and Pain Medicine, University of Washington and Seattle Children’s Hospital

**Abstract**: In order to effect lasting change, solutions to the pain epidemic must begin with prevention in childhood. The perioperative setting presents opportunity for prevention, with 15-20% of children presenting to pain clinics reporting surgery as a precipitating factor. A critical window of opportunity exists perioperatively to deliver interventions when families have a heightened state of attention, which can be optimized into learning coping skills. Indeed, psychosocial interventions effectively reduce acute postoperative pain. However these interventions typically are not offered to children having surgery, due to a lack of data regarding impact on longer term outcomes and due to a lack of access to psychosocial resources. To engage wide participation in a peri-operative program, interventions must use flexible, accessible, low-cost delivery models. Our research with family stakeholders indicates that families prefer technology-based delivery within the current system of perioperative care. mHealth is an innovative delivery approach which is easily integrated into clinical practice to address barriers related to socioeconomic status, and rural settings. These barriers led our team to develop SurgeryPal, an intervention delivered via internet and smartphone application teaching cognitive-behavioral based strategies to teens undergoing spine surgery and their parents. This scalable program is currently being tested in a multi-site HEAL effectiveness trial, and represents a significant advance in innovative approaches to deliver non-pharmacological therapies to youth undergoing major musculoskeletal surgeries. This intervention has potential to be integrated widely into pediatric surgery programs to prevent transition from acute to chronic pain.

**Concurrent Session Four**

**The role of the gut microbiome in somatosensation and pain - mechanisms and clinical implications**

**Session Chair**: Arkady Khoutorsky PhD, McGill University

**Session Abstract**: Changes in the gut microbiome have been recently observed in several chronic pain conditions, including visceral pain and chronic widespread pain. Accumulating evidence in human and animal studies suggests that these changes might contribute to the disease pathophysiology and enhanced pain sensitivity. The underlying molecular mechanisms, however, are poorly understood. In this session, we will overview the field of the gut microbiome in somatosensation and pain and discuss ongoing studies on the gut microbiome in different chronic pain conditions in humans. Specifically, we will present results supporting the causal role of the gut microbiome in fibromyalgia, providing evidence for: 1) alterations in gut microbiome composition and function in patients with fibromyalgia, and 2) the causal role of fibromyalgia-associated gut bacteria in causing pain hypersensitivity in animal models. We will discuss potential underlying mechanisms mediating the link between alterations in the microbiome and changes in neuronal activity and somatosensation, highlighting potential clinical implications of this new field, as well as its promising future directions.

**At the end of this presentation, participants will be able to**:

- Identify the importance of the microbiota in regulating sensory functions. They will gain a better understanding of the role of the microbiota on nociceptor specification and excitability and learn about the mechanisms by which the microbiota regulates nociceptor function.
- Develop a framework for understanding the role of gut microbiome metabolites in brain health and pain.
- Describe the role of gut microbiome in fibromyalgia in humans and animal models. Available measures of microbiome manipulation will be discussed. Attendees will also study the clinical effects of diet and fecal microbiome transplantation on symptom severity and the composition of the gut microbiome in women with fibromyalgia.

**Speaker One**

**Microbial colonization regulates nociceptor excitability and pain sensitivity in adulthood**

Christophe Altier

University of Calgary

**Abstract**: The gut microbiota regulates the development of the nervous system, thereby affecting sensory functions and host behaviors. The developmental time window of nociceptor specification coincides with microbial colonization and early life dysbiosis has been shown to impact nociceptor hypersensitivity in adulthood. How the microbiota influences nociceptor activity and pain sensitivity remain unknown. Methods: We have developed a germ-free (GF) TRPV1-GFP reporter mouse to isolate and phenotype TRPV1+ nociceptors in the absence of a microbiota. We determined the developmental regulation of nociceptors by bacterial colonization and the effects of commensal microbiota on pain sensitivity. We used Germ free-conventionalised TRPV1-GFP mice (colonized at postnatal day 21) and gnotobiotic TRPV1-GFP with stable defined moderately diverse microbiota mice (sDMDMm2) to identify bacterial species that govern nociceptor specification. Results: I will present our results showing that the microbiota regulates: 1) nociceptor sensitivity to noxious stimuli, 2) nociceptor molecular profiles and 3) nociceptor electrophysiological properties, and the overall impact of microbiota-mediated regulation of nociceptors on pain behaviors in adulthood. Our results highlight the importance of bacterial colonization in regulating the development and the function of nociceptors in adulthood. Our work will advance our understanding of the role of commensal bacteria in pain.

**Speaker Two**

**Gut microbiome metabolites in brain health and pain**

Shiqian Shen

Massachusetts General Hospital/Harvard Medical School

**Abstract**: Postoperative delirium (POD) occurs in 9-50% of older patients undergoing anesthesia and surgery but lacks reliable biomarkers and effective treatments. POD leads to worse clinical outcomes including higher mortality, and has become a pressing medical issue with significant public health relevance. Using liquid chromatography with tandem mass spectrometry (LC-MS)-based metabolomics technique, we recently revealed that baseline plasma levels of gut microbiota-related metabolite indole-3-propionic acid (IPA) were significantly negative-associated with the onset of POD in 103 (17 cases) human individuals. This association was validated in preclinical mouse models for POD: reducing IPA levels through gut microbiota perturbation promoted POD-like behavior. More importantly, IPA administration deterred POD-like behavior. Colonization of germ-free mice with mutant Clostridium sporogenes that did not produce IPA promoted POD-like behavior. The protective effect of IPA in mice was mediated, in part, by peroxisome proliferator-activated receptor gamma coactivator 1-alpha (PGC-1α) in hippocampalinterneurons. More interestingly, PGC-1α is a master regulator of mitochondria biogenesis which has been previously implicated in pain chronification. We are currently examining microbiome-derived IPA in neuropathic pain.

**Speaker Three**

**Gut microbiome manipulation as a potential therapeutic measure in fibromyalgia**

Amir Minerbi

Institute of Pain Medicine, Rambam Health Campus

**Abstract**: The gut microbiota consists of a diverse and dynamic community of microorganisms that inhabit the gastrointestinal tract and plays a role in host health and disease. Dysregulation of the gut microbial community has been linked to intestinal, metabolic, neurological, and psychiatric disorders. Fibromyalgia is a chronic pain disorder characterized by chronic widespread pain coupled with fatigue, sleep disturbances and cognitive dysfunction. We have recently demonstrated a substantial role for the gut microbiome in fibromyalgia: 1) In women with fibromyalgia, the composition of the gut microbiome is significantly altered; 2) Alteration of certain bacterial species in women with fibromyalgia is associated with changes in circulating metabolic end-products, including short-chain fatty acids and bile acids; 3) transplantation of gut microbiome from women with fibromyalgia, but not healthy controls, to germ-free mice induces pain hypersensitivity, which is reversible with antibiotics and healthy gutmicrobiome transplantation. We tested the potential utility of gut microbiome manipulation as a therapeutic modality in fibromyalgia by using 1) dietary interventions; and 2) fecal microbiome transplantation. First, we conducted a randomized clinical trial testing the clinical effects of low- FODMAP, gluten-free and usual diets on a cohort of 50 women with fibromyalgia while also evaluating the association of baseline gut microbiome composition and diet-induced alterations in gut bacteria with the clinical efficacy. Then, we performed a pilot study of fecal microbiome transplantation from healthy donors to twelve women with resistant fibromyalgia. Results of these studies will be presented, and the potential of gut-microbiome manipulation as a therapeutic measure in fibromyalgia will be discussed.

**Pain experiences of youth with neurodevelopmental disabilities (NDDs): Bridging research and practice**

**Session Chair**: Samantha Noyek MSc PhD, University of Calgary

**Session Abstract**: Globally, over 15% of the population lives with neurodevelopmental disabilities (NDDs). Children with NDDs are more likely to experience pain than their neurotypical peers, yet their pain is more likely to be overlooked and/or misinterpreted. This session unites research exploring pain assessment/management of NDD patient populations and emphasizes the importance of individual pain experiences of youth regardless of their diagnosis. This multidisciplinary session with international speakers (UK and Canada) will explore quantitative and qualitative research on pain assessment, responsivity, experience, and management in NDDs. Speaker one will characterize pain experiences (i.e., intensity, frequency, location, etc.) in youth with cerebral palsy (CP), and identify associations between mental health, pain outcomes, and quality of life. Speaker two will describe methods to explore perception of controlled responses to psychophysical pain induction in autistic youth, qualitative interviews with autistic children, and efficacy of pain management in this group. Speaker three will identify practices for pain assessment/management training for respite care providers of youth with intellectual disabilities, describe stakeholder feedback on parent-respite provider communication tools, and caregiver perceptions of needle pain management guidelines. An interactive discussion involving the speakers, a lived experience expert (via Zoom), and the audience, will consider i) individual differences in NDD populations and NDD abilities contributing to their pain assessment, ii) challenges in pain management/assessment, iii) approaches to bridge stakeholders and disciplines to address pain in NDDs. This session advocates for the pain experiences of youth with NDDs, to drive forward research priorities, clinical practice, and improve their quality of life.

**At the end of this presentation, participants will be able to**:

- Describe the current status and innovations of pain research within basic science, psychology, and health disciplines across different populations of youth with NDDs.
- Gain knowledge of the challenges to pain assessment, management, and efficacy of pain management in youth with NDDs.
- Gather insights from persons with lived experience and contribute to an engaged discussion of how stakeholders can contribute to a more holistic understanding of pain and mental health in youth with NDDs.

**Speaker One**

**The characterization of pain in children and youth with cerebral palsy: A micro-longitudinal analysis**

Carly Mc Morris

University of Calgary

**Abstract**: Background. Pain is present in up to 75% of children and youth with cerebral palsy (CP), making it the most prevalent secondary condition. Yet, little is known about the multiple dimensions of pain, the role of mental health in the experience of pain, and the impact of pain on quality of life in youth with CP. Further, no study has investigated how pain interferes with daily functioning using an Ecological Momentary Assessment approach. Method. Forty-three youth with CP (61.2% male; Mage = 11.66; SD = 2.77) completed cross-sectional measures of pain, mental health, and quality of life. Following, children completed 7 days of daily surveys measuring daily pain and functioning (i.e., “How much did pain interfere with your day?”). Preliminary Results. Youth reported a mean pain intensity of 3.70/10 and an average pain interference T-score of 52.25. Daily pain was endorsed by 23.3% of youth, and 46.3% reported pain location in their legs. Additionally, 51.2% of youth reported that they experience chronic pain. Pain interference and pain intensity were significantly correlated with anxiety, depression, and quality of life. Higher pain interference and pain intensity scores were associated with higher anxiety, higher depression, and a worse quality of life (p’s <.05). Discussion. This is the first study to characterize the pain experience in youth with CP. Findings will directly inform treatment-tailoring approaches to improve outcomes for this vulnerable population, and offset a trajectory of increased mental health issues, reduced quality of life, and poor functioning and disability into adulthood.

**Speaker Two**

**Pain in autistic young people**

David Moore

Liverpool John Moore University

**Abstract**: DSM-V criteria for autism include insensitivity to pain as a specific example of sensory atypicality. However, minimal research evidence exists to support this claim. The suggestion of altered pain responsivity in autism is distressing as pain may be the first or only sign of illness/injury and autistic people may be at greater risk of injury/illness than their peers. Autistic people might be more vulnerable to pain due to differences in communication and perceptions of being marginalised from the neurotypical healthcare system. Within our lab we are exploring pain within paediatric autistic populations, using methods designed to explore the perception of controlled responses to psychophysical pain induction, the neural correlates of responses, and individual differences in pain experience. We report the findings of recent co-produced interview studies with autistic children exploring how they experience daily pain. Here we explore how autistic young people experience pains ranging from headaches and abdominal cramps to falls and injuries. We discuss how young people disclose pain to trusted others, how they interact with primary healthcare and how they seek to manage their own pain. Finally, as we have observed an increased prevalence of autistic adolescents requiring treatment within tertiary chronic pain services, we explore the experiences of pain management and efficacy of pain management for autistic adolescents. In this session I will provide evidence that pain management needs to be carefully considered to support autistic people living with pain. It is necessary to develop evidence-based recommendations going forward for reasonable application to support autistic people.

**Speaker Three**

**One size doesn’t fit all: Tailoring interventions for individuals with intellectual and neurodiversity**

C. Meghan McMurtry

University of Guelph

**Abstract**: Individuals with intellectual and developmental disabilities frequently experience both acute and chronic pain but are at an increased risk of having their pain unrecognized and undermanaged. There is an abundance of literature on the management of acute and chronic pain in neurotypical populations, but less attention has been paid to neurodiverse populations. This is an equity problem. Furthermore, it is unclear whether gold standard approaches (e.g., existing clinical practice guidelines) based on neurotypical populations are familiar to, accessible, interpretable, and actionable by caregivers of these individuals. This talk will touch on three qualitative and quantitative data sets related to these gaps. Firstly, an overview of the rationale for and creation of pain assessment and management training for respite care providers caring for individual with intellectual and developmental disabilities will be provided; secondly, their perceptions of the value of the targeted pain training within a randomized control trial will be explored. Thirdly, the creation of and stakeholder feedback about a parent-respite provider communication tool (“The Caregiver Pain Information Guide”) will be discussed. Finally, the perceptions of caregivers of autistic youth about strategies from needle pain and fear management guidelines will be reviewed via both qualitative content analysis of caregiver interviews and their quantitative ratings. Discussion will focus on commonalities seen in how to tailor interventions to manage pain in individuals with intellectual and neurodiversity.

**Hot Topics Trainee Presentations**

**At the end of this session, participants will be able to**:

- Describe the latest research in pain mechanisms and clinical care; and
- Critique and evaluate emerging topics in pain research.

**Poster presentation: The comparative effectiveness of CGRP monoclonal antibody therapies and other pharmacotherapies for migraine prevention: A systematic review and network meta-analysis**

Tanvir Jassal

McMaster University

**Introduction/Aim**: There is limited evidence addressing the comparative effectiveness of drugs for migraine prophylaxis. We conducted a systematic review and network meta-analysis to facilitate comparison between drugs.

**Methods**: We searched MEDLINE, EMBASE, CENTRAL, and clinicaltrials.gov for randomized trials of pharmacologic treatments for migraine prophylaxis. Reviewers worked independently and in duplicate to screen references, extract data, and assess risk of bias. We performed a frequentist network meta-analysis and assessed the certainty (quality) of evidence using the GRADE minimal contextualized approach.

**Results**: We found fremanezumab, eptinezumab, erenumab, and topiramate resulted in an important reduction in monthly migraine days compared to placebo (high certainty). Galcanezumab, calcitonin gene receptor peptide (CGRP) receptor antagonists, beta-blockers, valproate, amitriptyline probably reduced monthly migraine days (moderate certainty). Carisbamate and oxcarbazepine demonstrated the same effect as placebo (high certainty). Gabapentin may be no different than placebo (low certainty) and we are very uncertain of the effects of calcium channel blockers (very low certainty).

For adverse events leading to discontinuation, fremanezumab, eptinezumab, erenumab, galcanezumab, and CGRP receptor antagonists probably do not increase adverse events leading to discontinuation and are well tolerated (moderate to high certainty). Fremanezumab, eptinezumab, galcanezumab, CGRP receptor blocker, and carisbamate were probably no different than placebo (moderate certainty). Valproate and topiramate increased adverse events leading to discontinuation, compared to placebo (high certainty).

**Discussion/Conclusions**: Monoclonal antibodies are the most effective for migraine prophylaxis with the best safety profile compared to older pharmacotherapies.

**Poster presentation: Selective lesion of claustro-cortical circuitry enhances pain**

Christian Faig

University of Alberta

**Introduction/Aim**: Pain perception is processed by a network of cortical structures which are disrupted in chronic pain. The claustrum (CLA), a subcortical nucleus, provides inhibitory control over this network through preferential recruitment of inhibitory interneurons. The function of this region remains unknown. The CLA reciprocally communicates with cortical and subcortical pain structures including the anterior cingulate cortex, indicating potential pain related functions. In this study, we hypothesize that nociception activates CLA projection neurons, which restricts cortical activity. Furthermore, claustrocortical activity is lost in chronic pain, driving hyperactivity of cortical pain structures.

**Methods**: Complete Freund’s adjuvant (CFA) or saline was injected in the hindpaw of male and female C57Bl/6J mice. Postmortem cFOS histology was used to determine which claustrum neurons are active in acute and chronic pain. A combinatorial viral vector strategy was used to induce Cre-driven expression of caspase to lesion claustrocortical projection neurons. The impact on sensory thresholds and spontaneous pain behaviour was assessed.

**Results**: Acute and persistent pain increased cFOS expression in CLA projection neurons. Selective viral lesion of claustrocortical projections lowered mechanical and thermal nociceptive thresholds compared to intact controls. CLA lesion did not impact chronic pain induced hypersensitivity but expedited onset.

**Discussion/Conclusions**: These results show that the claustrum is engaged by nociceptive stimuli and loss of claustrocortical neurons amplify pain behavior. This study identifies a novel circuit in the cortical pain network and deepens our understanding of how pain is generated and processed in the brain.

**Poster presentation: Fecal microbiota transplantation from fibromyalgia patients causes pain in mice**

Weihua Cai

McGill University

**Introduction/Aim**: Fibromyalgia is a chronic pain syndrome characterized by chronic widespread pain, accompanied by fatigue, sleep disturbances and cognitive dysfunction. There are no effective treatments available due to limited knowledge of the disease’s pathophysiology. In 2019, it has been discovered that the gut bacterial community composition is altered in fibromyalgia patients. Changes in the composition of the microbiota are found in many physiologic processes and various pathologies. This study aimed to explore whether the altered gut bacterial community composition plays a causal role in the development of fibromyalgia syndrome.

**Methods**: Fecal microbiota transplantation was performed to manipulate the microbiota in germ-free mice or antibiotics-treated mice. The changes in recipient mice’s phenotypes were tested by von Frey, Hargreaves, cold plate, mouse grimace scales, running wheels, novel objective location and tail suspension tests. The molecular changes were measured by immunofluorescence, RNA sequencing, metabolomics analysis and single-cell RNA sequencing.

**Results**: Colonization of germ-free mice with the gut microbiota from individuals with fibromyalgia caused pain hypersensitivity and reduced general activity. The colonization also induced depression-like behaviour at chronic but not acute time points. Fibromyalgia microbiota altered bile acids metabolism and immune landscape and induced spinal microglia changes in the recipient mice. Depletion of microglia partially alleviated the development of pain hypersensitivity. Remarkably, re-colonization of fibromyalgia recipient mice with the gut microbiota from healthy donors reversed pain hypersensitivity.

**Discussion/Conclusions**: These results demonstrate that the gut microbiota from individuals with fibromyalgia is sufficient to cause pain in mice, suggesting the potential role of altered microbial communities in mediating distinct phenotypes in fibromyalgia patients.

**Poster presentation: Vapourized cannabis transiently alleviates neuropathic pain and modulates spinal microglia activity in male rat**

Nynke J. van den Hoogen

University of Calgary

**Introduction/Aim**: Neuropathic pain is caused by injury or disease of the nervous system and is often resistant to analgesics. To alleviate pain symptoms, some patients use medical cannabis. However, the efficacy of cannabis for neuropathic pain is not well defined.

**Methods**: In this study, we assessed the impact of vapourized cannabis exposure on mechanical allodynia in a rat model of spared nerve injury (SNI). Adult Sprague-Dawley rats underwent SNI surgery, and were exposed to a novel in-cage vapourized cannabis exposure paradigm at 7 and 14 days post-SNI.

**Results**: At both 7 and 14 days both SNI, we determined that vapourized cannabis exposure attenuated allodynia up to 180 minutes. Amongst the extracts tested, a combination of 10% delta-9-Tetrahydrocannabinol (THC) and 10% cannabidiol (CBD), as well as a whole cannabis extract (containing both THC and CBD) was most effective at reducing allodynia. In addition, the number of cells containing microglia markers CD11b and CD68, marking general microglia activity and their pro-inflammatory phenotype, were reduced.

**Discussion/Conclusions**: Combined, our data show that vaporized cannabis acutely and transiently reduces mechanical allodynia, possibly through reducing microglial activation.

**Poster presentation: Parental validation in parent-child pain communication about pediatric chronic pain**

Maria Pavlova

University of Calgary

**Introduction/Aim**: Pediatric chronic pain is a disabling and costly condition that affects 15 to 40% of youth and impacts functioning of the whole family. Growing evidence demonstrates that pain communication plays an important role in pain experiences and outcomes. Validation (i.e., acceptance/confirmation that another’s pain is distressing and legitimate) in pain communication is emerging as one of the key aspects of pain communication influencing pain experiences. Validation, as opposed to invalidation, in pain communication leads to more positive affect and less worry, anger, and frustration. No studies have examined validation in parent-child pain communication. We aim to investigate the levels and predictors of parental validation in parent-child pain communication.

**Methods**: Forty youth aged 8 to 18 years with migraine and recurring attacks and one of their parents were recruited from Headache/Neurology clinics at a tertiary children’s hospital. Parents reported sociodemographic information. Participants completed a narrative elicitation task using a video-conferencing platform. Specifically, participants reminisced about two past attacks of migraine and a past event that involved sadness. The narratives coded using an established Validation and Invalidation Behavioral Coding System.

**Results**: Preliminary analyses revealed that parents were more validating when discussing past sad experiences, as compared to past attacks of migraine. Parents of younger, versus older, youth and parents who lived with chronic pain validated their children more frequently when discussing pain.

**Discussion/Conclusions**: Given the prevalence and economic burden of pediatric chronic pain, as well as the emerging importance of pain communication, further understanding of parent validation and its influence on pain outcomes is needed.

**Poster presentation: The interaction between adverse childhood experiences and pain predicts the severity of suicidality in youth over time**

Perri Tutelman

University of Calgary

**Introduction/Aim**: Youth with chronic pain who have experienced a greater numbers of adverse childhood experiences (ACEs) report higher levels of anxiety and depression and worse quality-of-life. However, little is known about the relationship between ACEs and pain in community samples and the risk they confer for more serious mental health issues, such as suicidality. This study examined the prospective relationship between ACEs and pain in the development of suicidality severity.

**Methods**: Participants were 139 healthy youth (Mage = 13.74 years, SD = 1.56, 64% female) between the ages of 11-17 years, recruited based on parental history of depression or anxiety. Youth completed validated measures of internalizing symptoms, ACEs, and pain characteristics at baseline and follow-up diagnostic interviews 9- and/or 18-months later to assess for severity of suicidality.

**Results**: After controlling for demographics, baseline internalizing symptoms, and ACEs, increased pain interference at baseline predicted increased suicidality severity at follow-up. Greater number of ACEs reported at baseline predicted increased suicidality severity at follow-up in youth with (b = 8.49, p = .003) but not without chronic pain (b = 2.19, p = .23) and in youth with high (b = 7.65, p < .0001) but not low (b = -3.38, p = .11) levels of pain interference and high (b = 6.96, p = .0003) but not low (b = .75, p = .74) levels of pain severity.

**Discussion/Conclusions**: The effect of chronic pain on suicidality may be potentiated by ACEs, such that elevated pain symptomology was only related to suicidality in children who experienced increased childhood adverse events. Results emphasise the importance of assessing for both ACEs and pain when screening for suicidality in community samples of youth.

**The intersection of pain and addiction: Using harm reduction strategies to keep patients with chronic pain safe within the context of the current opioid crisis**

**Session Chair**: Hance Clarke MD PhD, Toronto General Hospital

**Session Abstract**: The opioid crisis continues to be front and center in practitioners’ minds when making decisions regarding clinical care of chronic pain patients. However, practices that were influenced either directly or indirectly by guidelines that were intended to protect patient safety, such as opioid tapering, have also increased risks of harm. Canada continues to face significant challenges in supporting persons living with pain and who may also concurrently have a co-existing opioid use disorder. Introducing harm reduction practices into the chronic pain treatment paradigm is an absolute must to protect patient safety. There have been multiple studies demonstrating harms associated with abrupt tapering of the chronic pain patient. While harm reduction strategies are nationally supported and grow in primary care settings, hospitals demonstrate structural, policy-based and operational element deficiencies for the same. Patients suffering from opioid use disorder requiring acute care interventions (i.e. surgery) have reported feeling stigmatized when accessing health services. Often, many choose to leave hospital, further negatively affecting their health outcomes. This symposium will discuss the human rights of patients as they struggle to navigate the current chronic pain landscape. Second, this symposium will discuss how the Pain Program at Toronto General Hospital has started to identify methods through which harm reduction strategies have been explored for patients in acute phase of illness. Finally, will share a well-oiled mode of integrating pain addiction practices within the pain setting from St. Paul’s hospital in Vancouver - the epicentre of the opioid crisis.

**At the end of this presentation, participants will be able to**:

- Acquire a nuanced understanding of the impact of prescribing guidelines on the current medico-legal landscape and the human rights of patients.
- Define patient-centered application of harm reduction during hospitalization.
- Explore how pain and addiction medicine services can co-exist to improve patient outcomes.

**Speaker One**

**The rights of the patient suffering with chronic pain**

Kate Nicholson

National Advocacy Center

**Abstract**: Kate Nicholson, JD, is a U.S.-based human rights attorney and a person with lived experience of pain. Nicholson will provide a historical, legal, and ethical overview of a pendulum swing on opioid prescribing and its impact on the human rights of people living with pain. She will first discuss how intersecting prescribing guidelines in the U.S. and Canada have shaped the current medico-legal environment and have affected the human rights of patients. Next, she will present the most recent data on tapering and patient abandonment. In addressing this data, she will show how applying harm reduction principles to the treatment of patients can improve outcomes. Finally, she will lay the foundation for the other panelists by addressing why pain and addiction are not binary issues, but rather must be considered on a spectrum in the provision of patient-centered care, and how harm reduction provides a useful lens across all such patients. Rather than being a place where stigma, abandonment, and human rights violations occur, the healthcare system can and should be a critical point of opportunity to improve patient outcomes at a moment when the drug poisoning crisis is increasingly lethal.

**Speaker Two**

**Balancing perioperative pain management and supporting harm reduction**

Salima Ladak

Toronto General Hospital

**Abstract**: Despite the growing call for structured processes and systems to address their needs, patients with substance use disorder who require urgent hospitalization for a parallel health condition continue to face challenges in hospital. They have describe feeling stigmatized by health systems, and are in an often unprepared acute care setting to address harm reduction. Results of this experience often have negative consequences including self-discharge from hospital, reduced trust in health systems, a negative impact on individuals’ mental health as well as a lack of treatment for on-going health problems. Dr. Ladak will share patient centered harm-reduction approaches implemented for patients during acute illness. These patients were at significant risk of leaving hospital against medical advice. She will describe patients’ perspectives related their experience of pain relief, approach to harm reduction advice and intervention, as well as factors influencing their decision to almost self-discharge against medical advice or stop medical treatment. She will describe success factors resulting in sustained engagement of both health professionals and patients to ensure completion of required treatment for the patients’ primary health problems. Essential to this was the co-creation of clinical plans which included the patients, family and health providers. Community based harm reduction programs to help sustain patient engagement were also identified during hospitalization. Following these experiences of the Pain Program at Toronto General Hospital, this session will also describe hospital based changes that are being explored for improved future patient experience.

**Speaker Three**

**Perioperative pain management in the heart of the opioid crisis**

Ainsley Sutherland

St. Paul’s Hospital

**Session Abstract**: St. Paul’s Hospital in Vancouver is at the epicentre of the opioid crisis in Canada. A great proportion of the patients we care for come from marginalized populations and suffer from opioid use disorder, polysubstance use disorder and/or co-morbid psychiatric conditions. They have complex needs that require multidisciplinary care. These patients may present for elective procedures, traumatic injuries requiring surgery or medical complications of their substance use requiring surgery. These patient are often extremely opioid tolerant and may also be experiencing opioid induced hyperalgesia, further sensitizing them to the pain of surgery. High dose opioids, while required to prevent withdrawal and keep the patient from leaving hospital, may not be that helpful with managing acute pain. We are fortunate to have a large and well-established Addiction Medicine Consult Team at St. Paul’s who sees all patients with an active substance use disorder and works collaboratively with our Acute Pain Service to co-manage these patients. Multi-modal analgesia, including perineural catheters, epidurals, ketamine infusions and lidocaine infusions on the ward, and dexmedetomidine infusions in the high acuity unit may be used to help manage pain. Often AMCT takes the lead in managing the opioids - ordering high dose kadian, IV hydromorphone and/or methadone. APS manages the other aspects of pain management. On site, there is also a safe injection site where patients can use their drug of choice. The shared goal is to meet patients where they’re at in order to provide them with the care that they need.

**Concurrent Session Five**

**Pain and time**

**Session Chair**: Luda Diatchenko MD PhD, McGill University

**Session Abstract**: The molecular pathophysiology of chronic pain states is largely unknown. The genetic and molecular studies of pain in both humans and mice nevertheless can provide critical insights into pathophysiological mechanisms of pain chronification. Increasing evidence suggests that the resolution of acute pain requires an active molecular process that develops over time and not in a linear fashion. Failure of this resolution process will cause chronic pain. In this session, we will discuss the crucial importance of timing in the molecular events related to pain. Our findings suggest that this process is impaired in those who do not resolve acute pain over time and suggest time stratification of a cascade of processes resulting in a return to a normal, no-pain state. Dr. Diatchenko will illustrate this concept using blood samples of patients with acute low back pain. Dr. Ji will discuss the role of specialized pro-resolving mediators and their receptors in inflammatory and neuropathic pain in animals via neuro-immune interactions. Dr. Mogil will discuss the increasing evidence that important biological processes underlie chronic pain beginning at time points long after almost all existing preclinical pain studies are terminated.

**At the end of this presentation, participants will be able to**:

- Interpret the evidence of the dynamics of active biological processes that underlies pain resolution.
- Discover how pro-resolving mediators and their receptors regulate the resolution processes in immune/glial cells and neurons.
- Explore how pain-relevant biological changes occurring many months after injury.

**Speaker One**

**Acute inflammatory response via neutrophil activation protects against pain chronification**

Luda Diatchenko

McGill University

**Abstract**: The transition of acute to chronic pain represents a critical point of medical intervention. Here, I will report the investigation of the pathophysiological mechanisms underlying the transition from acute to chronic low back pain (LBP) at the transcriptome-wide level in peripheral immune cells. Transcriptomic changes were compared between patients whose LBP was resolved at three months with those whose LBP persisted. We found thousands of dynamic transcriptional changes over three months in LBP participants with resolved pain but none in those with persistent pain. The most prominent process that has been captured in this patient population was neutrophil-driven upregulation of inflammatory responses in an acute pain state. Importantly, this inflammatory response was substantial but transient, and down-regulated within three months of the observation period. In mouse pain assays, early treatment with a steroidal or non-steroidal anti-inflammatory drug (NSAID) also led to prolonged pain despite being analgesic short term; such a prolongation was not observed with other analgesics. Depletion of neutrophils greatly delayed the resolution of pain in mice while peripheral injection of neutrophils themselves prevented the development of long-lasting pain induced by an anti-inflammatory drug. Together, our results demonstrated the opposite contribution of neutrophil-driven inflammation to acute and chronic pain states and suggest the importance of prolonged observational time in animal experiments and clinical trials targeting drug development for chronic pain.

**Speaker Two**

**Resolution of pathological pain via SPMs and neuroimmune regulation**

Ru-Rong Ji

Duke University

**Abstract**: Increasing evidence suggests that resolution of actue pain requires an active molecular process and production of specilized pro-resolving mediators (SPMs), including resolvins, protectins, maresins, and lipoxins. A key mechanism for SPMs to resolve inflammation is to promote macrophage phagocytosis of the damaged and apoptotic cells. Neuroprotectin D1 (NPD1) is a SPM derived from fish oil DHA and possess potent anti-inflammatory and pro-resolution actions in animal models. NPD1 produces potent pain relief in animal models of inflammatory, neuropathic, and cancer pain. We recently identified the orphan receptor GPR37 as a novel receptor for NPD1. GPR37 is expressed by macrophages, DRG neurons, and oligodendrocytes in the spinal cord and brain. Notably, *Gpr37* knockout mice show deficits in resolving inflammatory pain and infection-induced pain. GPR37 regulates the resolution of pathological pain by altering macrophage M1/M2 phenotype and promoting macrophage phagocytosis. GPR37 agonists control inflammation and protect against septic dealth in animal models of infections. I will also discuss other mechanisms of SPMs for the resolution of pain, including modulation of TRP channels, synaptic plasticity, and glial activation. Together, these findings suggest that SPM-based proresolution pathways control the resolution of pain and inflammation via immune, glial, and neuronal regulations.

**Speaker Three**

**When does pain actually “transition” from acute to chronic?**

Jeffrey Mogil

McGill University

**Abstract**: The pain field has coalesced in recent years around a rubric suggesting that chronic pain is caused, in those who develop it, by the “transitioning” of acute into chronic pain. This talk will examine the evidence for this proposition, in both humans and animals. If there is a transition to chronic pain in laboratory animals, when does it occur. Evidence will be presented that a number of biological events both maintaining pain itself (e.g., cellular senescence, abnormal end organ targeting) and responsible for pain-related comorbidities (e.g., anxiety/depression, mortality) occur at much later time points (i.e., months after injury) than are currently represented in most extant preclinical pain research. This evidence not only suggests that pain research experiments should last much longer than they do, but also suggests a reexamination of the concept of “time” in species with different life spans.

**Neurophysiological changes following physical or emotional childhood trauma in the transition from acute to chronic pain**

**Session Chair**: Jillian Miller PhD, University of Calgary

**Session Abstract**: Pain has often been the first indication to me that something was not quite right,” (Taylor Pigott, PWLE and Medical Student, University of Calgary). However, there is emerging research to demonstrate that there are neurophysiological changes that precede and may contribute to the development of chronic pain in youth. Physical and/or emotional traumas may initiate a cascade of physiological stress, leading to structural and functional changes within the brain, which can modify perception and responses to pain. Taylor will speak to her personal journey dealing with skeletal malformations, fibromyalgia, and mental health. Dr. Julie Christianson (Professor in Cell Biology and Physiology, University of Kansas Medical Center) will present on early life stress and its impact on hippocampal development via changes in neurogenesis, gene expression, and DNA methylation. Samantha Miller (MSc Student in Medical Science, University of Calgary) will present the results from her Undergraduate Honours Thesis demonstrating the relationships between trauma, functional brain changes, self-report of pain, and pain responses to mechanical and thermal stimuli in a community sample of youth. And Dr. Sarah Nelson (Assistant Professor in Psychiatry and Behavioral Sciences, Harvard Medical School) will share her work examining adverse childhood experiences (ACEs; e.g., abuse, neglect), and biomarkers for allostatic load, as a potential contributor to pain maintenance in youth with and without chronic pain. Across all three studies, which include rodents, healthy and clinical samples of youth there is involvement of stress-related brain regions and systems, underlying changes in pain expression, highlighting important therapeutic targets for intervention.

**At the end of this presentation, participants will be able to**:

- Discuss how early life stress impacts the development of the hippocampus through changes in neurogenesis, gene expression, and DNA methylation, in a clinically relevant model of urological chronic pelvic pain syndrome.
- Demonstrate how exposure to trauma may alter brain functioning and modify pain thresholds, to potentially increase the risk of developing chronic pain in a non-clinical sample of adolescents.
- Identify how the physiological stress response may manifest in youth with chronic pain and potentially contribute to pain chronicity.

**Speaker One**

**The waiting game: Using advanced mri to understand the neurobiological transition from acute to chronic pain in adolescent rats**

Richelle Mychasiuk

Monash University

**Abstract**: Persistent post-surgical pain affects 20% of youth undergoing a surgical procedure, with females exhibiting increased prevalence of chronic pain when compared to males. Given this high prevalence of persistent pain we sought to examine sexually-dimorphic neurobiological changes underlying the transition from acute to chronic pain following surgery in adolescence. Male and female Sprague Dawley rats were randomly allocated to a sham or injury condition and assessed for pain sensitivity while also undergoing magnetic resonance imaging (MRI) at both an acute and chronic timepoint, both being completed within adolescence. We found that injury resulted in persistent pain in both sexes, with females displaying greater sensitivity. Total grey matter density was increased at the chronic timepoint in the female injured group, and injury altered grey matter density in brain regions such as the cerebellum, with female driven changes in the amygdala, insula, and caudate putamen, and male driven changes in the hippocampus and thalamus. Differences between chronic and acute density in the cerebellum and periaqueductal gray were directly correlated with change in pain sensitivity across time. Overall, our results indicate persistent behavioural and neurobiological changes following surgery in adolescence, with sexually-dimorphic and age-specific outcomes, highlighting the importance of studying both sexes and adolescents, rather than extrapolating from adult male literature.

**Speaker Two**

**Impact of early life trauma on brain efficiency and the development of pain symptoms in youth**

Samantha Miller

University of Calgary

**Abstract**: Both chronic pain (pain > 3 months) and adverse childhood experiences (ACEs) have been found to disrupt brain function. However, the extent to which changes to shared neural networks underlie the relationship between chronic pain and trauma has yet to be determined. Healthy youth aged 14-18 years were recruited from the community. Resting-state functional MRI data was obtained, and functional connectivity was assessed using graph theory metrics. Exposure to ACEs and posttraumatic stress symptoms were measured using psychometrically sound questionnaires. Objective measures of pain sensitivity were obtained by determining thresholds for mechanical and thermal noxious stimuli. Data was collected at two time points, three months apart, to examine changes over time. Youth exposed to higher numbers of ACEs demonstrated reduced global and local brain efficiency, as compared to youth with fewer ACEs. The interaction between number of ACEs and whole-brain efficiency measures were associated with altered pain symptomology, such that greater exposure to ACEs and lower brain efficiency were related to increased pain intensity and higher thresholds for painful stimuli. Youth exposed to ACEs may be less efficient at processing information, and slower processing speeds may lead to higher pain thresholds. Further, trauma-exposed youth may experience dissociative symptoms, leading to higher pain thresholds and poorer prognosis. The results of this study are consistent with existing literature on youth with chronic pain, and support the notion that early life trauma may confer a greater risk of developing persistent pain.

**Speaker Three**

**Investigating adverse childhood experiences (ACEs) on a physiological level: How mechanisms of aces may contribute to pain chronicity in pediatric populations**

Sarah Nelson

Harvard Medical School

**Abstract**: Adverse childhood experiences (ACEs; e.g., abuse, neglect, parent/guardian separation or divorce, parent/guardian mental illness) and the ensuing physiological stress response can negatively impact the body in a multitude of ways, with youth being a particularly vulnerable period for maladaptive changes. Emerging research in youth with chronic pain suggest that these individuals experience ACEs at a disparately higher rate than the average population and, in parallel, that physiological mechanisms of pain (e.g., nociceptive processing) closely connect to the physiological stress response (e.g., HPA-axis functioning, allostatic load). However, minimal research has directly examined these mechanisms in youth with chronic pain. Therefore, pilot investigations were conducted into these mechanisms in a treatment seeking pediatric pain population, which highlight avenues for future research in this context. Specifically, pilot data on the multifactorial construct of allostatic load (i.e., physiological wear and tear to multiple regulatory systems) as a potential contributor to pain maintenance and on neuroimaging data in youth exposed to ACEs with and without chronic pain were employed. From this, a conceptual framework was developed identifying chronic pain as a potential stressor in and of itself, categorized as a source of *toxic stress*, which could serve to contribute to pain chronicity, particularly in marginalized groups (e.g., racial/ethnic minorities, gender diverse youth).

**Can clinical trials translate pain science into improved care for people suffering from pain? Incorporating basic principles with patient engagement and real-world clinical practice**

**Session Chair**: Ian Gilron MD MSc FRCPC, Queen’s University

**Session Abstract**: Rigorous evaluation of the safety and effectiveness of a new pain treatment starts with clinical trials carefully designed to compare the new intervention to a placebo, sham control, or active comparator. As Dr. Ian Gilron will discuss, the purpose of a trial (e.g. “first in human” vs. large-scale pragmatic trial) dictates the necessary balance between internal validity and generalizability and demands consideration of: 1) participant population; 2) dose, duration and delivery of the intervention and comparator; and 3) the outcomes of interest. Beyond an obviously critical role as trial participants, there has been a growing appreciation that persons with lived pain experience should be engaged with researchers and clinicians to better inform the design, conduct and interpretation of clinical trials. As Dr. Dawn Richards will review, recent efforts for patient engagement in clinical research have included supports related to capacity-building and enhancing the participant experience. Thoughtful review and consideration of collective clinical trial results in pain research has revealed several challenges to their interpretation and incorporation into clinical practice. To address these challenges, Dr. James Khan will provide an overview of important considerations in pain clinical trial development including a) importance of blinding in pain clinical trials; b) sample size, risks of small trials, and role for large-scale trials; c) considerations for interpretation of results; and d) suggestions for future directions. In depth consideration of these diverse issues will provide opportunities to accelerate the implementation of promising new pain treatments into improved patient care.

**At the end of this presentation, participants will be able to**:

- Describe various aspects of participant population, characteristics of the treatment intervention and outcomes of interest when balancing internal validity with generalizability in clinical trials of pain treatments.
- Identify new efforts whereby patient engagement is guiding recent innovations in the design, conduct and interpretation of clinical pain trials.
- Recognize challenges and opportunities associated with the interpretation of clinical trials of pain treatments and their implementation into clinical practice.

**Speaker One**

**Basic principles of analgesic clinical trials**

Ian Gilron

Queen’s University

**Abstract**: Rigorous evaluation of the safety and effectiveness of a new pain treatment starts with clinical trials carefully designed to compare the new intervention to a placebo, sham control, or active comparator. As Dr. Ian Gilron will discuss, the purpose of a trial (e.g. “first in human” vs. large-scale pragmatic trial) dictates the necessary balance between internal validity and generalizability and demands consideration of: 1) participant population; 2) dose, duration and delivery of the intervention and comparator; and 3) the outcomes of interest.

**Speaker Two**

**Patient engagement in clinical pain research**

Dawn Richards

Five02 Labs Inc.

**Abstract**: Beyond an obviously critical role as trial participants, there has been a growing appreciation that persons with lived pain experience should be engaged with researchers and clinicians to better inform the design, conduct and interpretation of clinical trials. As Dr. Dawn Richards will review, recent efforts for patient engagement in clinical research have included supports related to capacity-building and enhancing the participant experience.

**Speaker Three**

**Relevance of clinical trials to clinical practice: Challenges of interpretation and implementation**

James Khan

University of Toronto

**Abstract**: Thoughtful review and consideration of collective clinical trial results in pain research has revealed several challenges to their interpretation and incorporation into clinical practice. To address these challenges, Dr. James Khan will provide an overview of important considerations in pain clinical trial development including a) importance of blinding in pain clinical trials; b) sample size, risks of small trials, and role for large-scale trials; c) considerations for interpretation of results; and d) suggestions for future directions. In depth consideration of these diverse issues will provide opportunities to accelerate the implementation of promising new pain treatments into improved patient care.

**Exploring and healing pediatric chronic pain in indigenous youth and families through two-eyed seeing**

**Session Chair:** Fatima Di Valentin, MSW BSW, Children’s Hospital - London Health Sciences Centre Schulich School of Medicine and Dentistry

**Session Abstract**: Two-Eyed Seeing is an integrative science approach that refers to “learning to see from one eye with the strengths of Indigenous ways of knowing and from the other eye with the strengths of Western ways of knowing and to using both of these eyes together.” (Bartlett, Marshall, & Marshall, 2012, p. 335). Our clinical researchers recognized the need for this approach, due to the low percentage of self-identified Indigenous patients (2%) accessing specialized outpatient pediatric chronic pain care in Ontario. Indigenous youth have a higher prevalence of chronic pain, painful medical conditions, disease-related pain, and dental pain when compared to non-Indigenous youth. They are more likely not to be treated for their pain and are likely to experience barriers to pain management services (Latimer et al., 2012; Turk & Okifuji, 2002). The World Health Organization has declared that pain management is a basic human right, and yet Indigenous youth and families needlessly suffer from insufficiently treated pediatric chronic pain. In this series of talks, Two-Eyed Seeing was employed to 1. understand patient and caregiver experiences of chronic pain and treatment; 2., exemplify using a biopsychosocial-spiritual approach to chronic pain treatment, and 3. present a feasibility study of group treatments cocreated by Indigenous adolescent patients with chronic pain and their caregivers.

**At the end of this presentation, participants will be able to**:

- Explore expressions of pain and healing in Indigenous youth and caregivers.
- Exemplify Two-Eyed Seeing in clinical and research methodology (formerly provide a template for two eyed seeing clinical and research methodology.
- Report on an intervention cocreated by indigenous youth and their caregivers.

**Speaker One**

**Exploring pain expression, healthcare barriers, and preferred treatments of indigenous youth with pediatric chronic pain and their caregivers through art and story-telling**

Abirami Kandasamy

Children’s Hospital - London Health Sciences Centre Schulich School of Medicine & Dentistry

**Abstract**: Background: Indigenous children and their families, in Canada, have had a long history of barriers to accessing healthcare (Nguyen, Subhan, Williams, & Chan, 2020). Barriers can vary and include geographic factors, negative bias from healthcare providers, inaccessible health education systems, employment and income inequities, racism, and social exclusions (Nguyen et al., 2020). Accessing specialized chronic pain treatment is reliant on the clinical judgment of primary healthcare providers and is based on the provider’s interpretation of the patient’s physical, verbal, and narrative expression of pain (Earp et al., 2019). For Indigenous youth, cultural differences in pain expression, may result in missed diagnoses of chronic pain (Latimer et al., 2018). Art-making and story-telling is a cultural practice in many Indigenous communities. There is research that supports art as a means of measuring pain expression in Indigenous youth (Latimer et al., 2018). The objectives of this study were to explore adolescent and caregiver expressions of pain using different modalities such as art and narratives. Participants and Methods: Six Indigenous adolescents with pediatric chronic pain (ages 14 to 17) and their caregivers participated in art-therapy and narrative-based focus groups that explored their identity, the experiences of pain on an individual, family, and community level, barriers to treatment for chronic pain, and their preferred treatments. This focus group was cocreated and led by Indigenous healers. Results from this recently completed qualitative study will be presented. This study offers different methodologies for evaluating pain expression and provides insights into potential interventions to improve health equity for Indigenous families as well as treatment procedures that integrate spiritual and cultural practices.

**Speaker Two**

**Applying the biopsychosocial-spiritual model to an interdisciplinary treatment model for indigenous adolescents with pediatric chronic pain**

Jonathan Gregory

Children’s Hospital - London Health Sciences Centre Schulich School of Medicine & Dentistry

**Abstract**: Best practices for assessing and treating chronic pain are based on the biopsychosocial model (Wakefield and Jerson, 2017). The biopsychosocial model has been extended to incorporate spiritual components and Indigenous healing practices in efforts to make assessments and treatments for chronic pain more culturally responsive for Indigenous peoples. Specifically, the biopsychosocialspiritual model integrates practices to emphasize the influences of mind-body-spirt interconnections to heal chronic pain among Indigenous children, adolescents, and families (Mark & Lyons, 2014; Taylor et al., 2013). In this series of case studies, we report on using this model to take a Two-Eyed Seeing approach to clinical practice for all aspects of clinical care as designed by Indigenous healers and patient councils made up of Indigenous youth and caregivers. In this talk, we will discuss navigating integration of cultural and spiritual processes into interdisciplinary intakes, individual sessions with psychology, physiotherapy, and psychiatry, as well as family sessions with social work. We will also highlight partnering with traditional healers in the clinical process and present a case of a patient treated with Western medications as well as traditional Indigenous medicines for managing pediatric chronic pain. Ultimately, we hope that our case studies and clinical lessons learned will support interdisciplinary chronic pain programs to extend the existing treatment programs and move toward a holistic, culturally safe biopsychosocial-spiritual model.

**Speaker Three**

**Pilot evaluation of Mukwa medicine: Acceptability of a virtual cognitive behavioural pediatric chronic pain therapy for and by indigenous youth**

Maisha Syeda

Children’s Hospital - London Health Sciences Centre Schulich School of Medicine & Dentistry

**Abstract**: Individual and systematic adversities significantly increase risks for chronic pain among Indigenous adolescents. Additionally, reduced access to chronic pain treatments and related medical services and experiences of racism and oppression in the healthcare system exacerbates the impairments associated with chronic pain among Indigenous adolescents, reducing their quality of life. Virtual delivery of cognitive behavioural therapy (V-CBT) has consistently demonstrated efficacy, and early research on the adaptation V-CBT to pediatric chronic pain populations has shown promising outcomes. However, there is a paucity of culturally-responsive V-CBT chronic pain programs for Indigenous adolescents that integrate Indigenous teachings and practices. This presentation will showcase the pilot evaluation of “Mukwa Medicine: Healing Pediatric Pain” Group, a V-CBT-based program built on the Two-Eyes Seeing biopsychosocial-spiritual model. The novelty of “Mukwa Medicine” is that it offers: 1) personalized traditional healing practices, 2) an interdisciplinary virtual treatment (e.g., led by an Art Therapist and co-facilitated by a psychologist, integrating a nurse-facilitated pain neuroscience education and a physiotherapist-facilitated pacing and activity planning sessions), 3) integration with patient-council approved CBT for pain psychotherapy modules that include relaxation teaching; and 4) integration of mindfulness techniques (Palermo et al., 2010; Veehof et al., & Schreurs). Mukwa Medicine has both parent and adolescent psychotherapeutic components (i.e., two separate groups). It consists of 7 virtual group sessions and 2-in person booster sessions, and structured feedback was sought from a patient council comprising Indigenous adolescents living with chronic pain to enhance its cultural relevancy. We decided to implement the program virtually to improve accessibility and reach Indigenous adolescents facing geographical and financial barriers to attend in-person services. At the same time, the patient council recommended having in-person booster sessions to accommodate culturally specific healing and grounding practices (e.g., nature walks). Evaluation data is currently being collected for the pilot of Mukwa Medicine. Participants will be invited to participate in individual interviews to describe their experiences and provide feedback for changes and improvements as well as complete narratives and surveys pre-and-post-program to report on pain, functional ability, anxiety, and self-efficacy.

**Concurrent Session Six**

**Bridging the gap: Translational approaches to studying pain in rodent and human models**

**Session Chair:** Eve Tsai, MD PhD, The Ottawa Hospital, University of Ottawa, The Ottawa Hospital Research Institute

**Session Abstract**: To understand the molecular mechanisms that underlie pain, basic scientists must rely on animal models. Although critically important in the therapeutic-development pipeline, traditional animal models leave a translational gap that has contributed to high failure rates when findings from rodent preclinical models are developed directly into new pain therapies. In this symposia, Dr. Bryan Copits will discuss the use of human nervous system tissue to build a catalog of human cell types based on their functional properties, transcriptomic profiles and morphology. To bridge the gap between basic science pain studies and clinical applications, Dr. Katelyn Sadler will discuss her use of transgenic mice, patient samples, and human donor tissue to characterize compounds that drive chronic pain in sickle cell disease. Finally, Dr. Annemarie Dedek will share approaches to studying electrophysiological properties of individual neurons and spinal nociceptive circuits in rats and human donors. The Chair, Dr. Eve Tsai, will bring her expertise as a clinician scientist and neurosurgeon to lead a discussion on the preclinical approaches and translational potential for the findings discussed in each talk. Together, this symposium will inform on the latest approaches to closing the gap that exists between rodent preclinical models and the target human pain population.

**At the end of this presentation, participants will be able to**:

- Describe barriers that exist between rodent pain models and the clinical population.
- Formulate applications of translational preclinical human tissue models to study functional properties of pain-processing neurons.
- Develop strategies for pairing rodent pain models with preclinical human tissue applications.

**Speaker One**

**Electrophysiological approaches to studying spinal nociceptive circuitry in rats and humans**

Annemarie Dedek

Carleton University; The Ottawa Hospital Research Institute

**Abstract**: The superficial dorsal horn (SDH) of the spinal cord houses critical circuitry for processing nociception. Despite the importance of the SDH to understanding pain physiology, little is known about how functional properties of the SDH may differ by sex and between rats and humans. Uncovering potential differences between sex and species is critical for translating preclinical findings in rodents to clinical applications in humans. Here, I will discuss pairing whole-cell patch clamp recordings of individual SDH neurons with high-definition multi-electrode array recordings of spinal slices in rat and human tissue in both sexes. First, I will describe the pioneering development of these electrophysiological applications in human tissue models. Next, I will discuss if the molecular and functional properties of excitatory NMDA receptor synaptic responses are conserved by sex and species. By combining patch-clamp recordings of outward miniature excitatory postsynaptic currents with non-biased data analysis approaches, we uncovered a wide range of functional signatures of excitatory synaptic events within individual SDH neurons. This heterogeneity in properties of excitatory synaptic events within individual SDH neurons may enable synapse-specific forms of plasticity and sensory integration within dorsal horn nociceptive networks. Finally, I will conclude with our investigation of the potentiating effects that the pain-modifying peptides brain-derived neurotrophic factor (BDNF) and calcitonin gene-related peptide (CGRP) have on spinal circuity in both sexes of rats and humans. Together, these findings provide the first-ever direct electrophysiological comparison of spinal nociceptive circuity by sex, across rat and human samples.

**Speaker Two**

**Pain in the gut: Gastrointestinal chemosensation drives chronic pain in sickle cell disease**

Katelyn Sadler

Center for Advanced Pain Studies, University of Texas at Dallas

**Abstract**: Many patients with sickle cell disease (SCD) suffer from chronic pain, the underlying causes of which are unclear. Recent 16s ribosomal RNA gene sequencing studies revealed differences in the bacteria that colonize the gastrointestinal tract of patients and mouse models with SCD relative to healthy controls, but it is unclear if or how these changes contribute to SCD pain. In these experiments, we used transgenic mice, patient samples, and human donor tissue to characterize compounds that drive chronic SCD pain by altering visceral chemosensation. Using antibiotic, probiotic, and fecal material transplant manipulations, we first determined that contents of the SCD mouse gut contribute to the evoked pain behaviors exhibited by these animals. We then performed 16s rRNA gene sequencing and unbiased metabolomic screening to characterize dysregulated bacterial species and compounds that could be driving pain in SCD fecal material transplant recipients. We identified bilirubin as one such metabolite; bilirubin induced calcium flux when directly applied to mouse nodose ganglia neurons, increased nodose ganglia neuron excitability, and, when orally administered to wildtype mice, evoked vagus nerve-dependent pain behaviors and altered gut bacterial populations. To determine if this metabolite is also relevant for pain in patients with SCD, bilirubin activity was assessed in human dorsal root ganglia neurons and bilirubin levels were measured in patient serum at steady state and during an acute pain episode. These experiments provide a translational framework for future studies that will examine how bacterial species and metabolites alter host physiology in various chronic pain conditions.

**Speaker Three**

**Human nervous system tissue for preclinical pain research**

Bryan Copits

Pain Center, Washington University of School of Medicine, St. Louis

**Abstract**: Despite major advances in the understanding the genes, diverse cell types, and circuits for processing pain in model organisms, there has been little success in translating these discoveries into new treatments. One of the major hurdles to effective translation of research findings is the lack of detailed information on human biology. To address these barriers, we embarked on a new approach to incorporate human pain-related tissues into our research program to test whether species-specific differences could be hampering efforts to develop new therapeutics. In this talk, I will discuss our work to establish partnerships with local organ procurement organizations to incorporate human nervous system tissue into our research program. I will also outline steps that can be taken to establish similar collaborations at other institutions. We are currently using human sensory neurons and spinal cord tissue to build a catalog of human cell types defined by their functional properties, transcriptomic profiles, and neuronal morphology to enable more direct comparisons between humans and model organisms. This work also involves tissues from donors with chronic pain and opioid use disorders to understand how these diseases impact human neurophysiology and cellular identity. Our long-term goal is to identify unique molecular targets in the human nervous system that can be targeted for new therapies to treat these debilitating diseases.

**Mobile health data for pediatric pain: Inter-institutional experiences and best practices for patient-centred data capture, analysis, and visualization**

**Session Chair:** Vina Mohabir BSc (Hons), The Hospital for Sick Children

**Session Abstract**: Mobile health (“mHealth”) refers to the use of mobile phones and wearable devices to achieve improved health goals. Prominent mHealth applications include the longitudinal collection of subjective data using ecological momentary assessments and objective data related to sleep, activity and function using wearables.

In the context of pediatric pain, mHealth has been used successfully to collect dense, contemporaneous data to address complex research questions. However, applications of mHealth data to empower pediatric patients with insights about their painful condition are rare. It is not easy to distill potentially thousands of data points and present them back to pediatric patients in a form that is both meaningful and valuable. Striving to achieve this goal requires thoughtful involvement of patients in all stages of protocol design and execution.

Our research groups, based at large pediatric hospitals across North America, have been independently working in this space for over 5 years. In 2021, an interdisciplinary collaborative was struck to develop experience-based best practices for applying patient-focused mHealth methods to pediatric pain. In this symposium, we will share our experiences and offer guidance on how to collect, analyze, and visualize mHealth data for pediatric pain. Specific case applications of wearables and smartphone apps for acute and chronic pain settings will be highlighted. The session will be moderated by a person with lived pain experience, who will draw together key themes and recommendations for meaningful inclusion of youth voices into the design and implementation of data visualization processes.

**At the end of this presentation, participants will be able to**:

- Articulate the considerations and benefits of different types of mHealth data capture and visualizations for youth with acute and chronic pain.
- Analyze the advantages and disadvantages of various platform choices for mHealth data capture, analysis, and visualization.
- Formulate a roadmap for meaningful inclusion of youth voices across the developmental spectrum into the design and implementation of data capture and visualization processes.

**Speaker One**

**Co-design and implementation of smartphone-based symptom visualizations for youth with acute and chronic pain**

Chitra Lalloo

The Hospital for Sick Children; University of Toronto

**Abstract**: iCanCope with Pain is a smartphone-based self-management platform for youth living with painful conditions. Developed by The Hospital for Sick Children and University Health Network, the iCanCope platform has been adapted for youth with chronic pain, juvenile idiopathic arthritis, sickle cell disease, neurofibromatosis, and post-operative pain. Emerging evidence from clinical trials demonstrates high user satisfaction and positive impacts on pain-related outcomes.

Daily symptom tracking is a core feature of iCanCope. Designed in collaboration with patient partners, the app uses gamified Likert scales to capture daily “check-in” reports of pain intensity, activity limitations, mood, sleep, physical activity, and energy levels. While the primary purpose of iCanCope “check-ins” is to inform personalized recommendations for pain self-management strategies, youth also requested the provision of on-demand, interactive visualizations of their symptom data.

Dr. Lalloo will share her team’s experience with iterative development and testing of various data visualization modalities, including interactive heatmaps and trend graphs. She will describe considerations for optimizing practical value for users and data interpretability in collaboration with youth patient partners. Contextual factors, such as design adjustments for acute versus persistent pain conditions and accessibility needs, will be discussed. Finally, user-level analytics from multiple clinical trials will offer insights to how youth engage with the visualizations over time and qualitative data will illustrate youth experiences with the feature in their own words.

**Speaker Two**

**In-home data capture and visualization as an opportunity for enhancing equity in research and interventions for pediatric chronic pain**

Kateleynn Boerner

BC Children’s Hospital; The University of British Columbia

**Abstract**: The deployment of mobile health technologies has tremendous potential to increase accessibility to pain interventions. Data visualization in particular provides an opportunity to enhance engagement with inhome data collection, motivate self-management, and can act as an adjunct for the delivery of evidencebased pain interventions, such as cognitive-behavioural therapy. However, these platforms are not without risk; rigorous and thoughtful evaluation is needed to ensure that design is patient-centered, privacy is protected, and adverse events (e.g., focusing attention on pain) are minimized. MyWeekInSight is a platform that was co-designed with patients, clinicians, and computer scientists. It collects ecological momentary assessment (EMA) data regarding symptoms, sleep, functioning, social interactions, and emotions. This data is then visualized into graphs, heat maps, and charts that patients can view to reflect on the interactions between these factors and their overall pain and well-being. Dr. Boerner will share lessons learned from a pilot feasibility trial of MyWeekInSight data visualization as an intervention for pediatric chronic pain, using a randomized controlled cross-over design. This will be presented alongside data from a larger initiative, the Living Lab at Home (LLAH), co-led by Drs. Oberlander & Boerner at BC Children’s Hospital, that aims to increase accessibility of in-home multi-modality micro-longitudinal research for families across the developmental spectrum, specifically considering the adaptation of in-home data collection and visualization for youth with sensory, motor, and communication differences. Dr. Boerner will reflect on the implications of such mobile health platforms for addressing health inequities in the context of pediatric chronic pain.

**Speaker Three**

**Integrating smartphones and wearables in the clinical characterization of pediatric acute and chronic pain patients**

Joe Kossowsky

Boston Children’s Hospital; Harvard Medical School

**Abstract**: A longstanding barrier to progress in tailoring individual assessment and interventions in children and adolescents with acute and chronic pain has been the fundamental difficulty of accurately assessing and characterizing social and behavioral patterns in the patients’ natural setting, in a continuous, nonobtrusive and efficient fashion.

In his talk, Dr. Kossowsky will discuss his lab’s experience in capturing and visualizing patients’ social and behavioral patterns in inpatient and home settings, using a combination of ecological momentary assessment, wearables, digital cognitive tests and embedded sensors in smartphones. He will demonstrate how the data from these mobile health tools are integrated with the rich clinical data offered in hospital settings to provide insights and visualizations of mobility patterns, social patterns, sleep patterns and cognitive function as a stream of real time data in various pediatric pain populations.

Finally, he will present data and reflect on the attitudes, concerns and preferences of adolescent pain patients and their parents on the use of these mobile health tools in both inpatient and outpatient settings.

**Considering the multiple identities of patients with chronic pain: How can we tailor pain care to support young athletes?**

**Session Chairs:** Melanie Noel, University of Calgary

**Session Co-Chair:** Katya Dittrich, Person with lived experience

**Session Abstract**: Chronic pain is among the largest contributors to functional impairment in children globally, and response to treatment is suboptimal (~50% of patients refractory). Athletes are at particular risk for developing chronic pain as injury is a common incident in sport and has been established as a precipitating factor for chronic pain. Despite the risk and deleterious outcomes associated with chronic pain, limited research exists to guide treatment and management of pain for this specific population. Novel approaches to interdisciplinary intervention (e.g., graded exposure) which could support athletes who are navigating CP. For athletes, however, limited research exists to guide how this standard of treatment could be better integrated into a sport-specific context. Centered around the narrative of an athlete with lived experience of chronic pain in a high-level sport sphere, this symposium aims to build a bridge between pain treatment and sport. To do this, the authors will: (a) characterize a population of youth athletes diagnosed with chronic pain, including salient demographic and psychosocial characteristics, (b) describe current treatment approaches for the rehabilitation for chronic pain, highlighting unique considerations and adaptations to established interventions that might be important for athletes, and (c) present a conceptual framework for interprofessional collaboration between pain and sport spheres so to improve outcomes for youth athletes with chronic pain.

**At the end of this presentation, participants will be able to**:

- Identify the impact chronic pain can have on a high-performance, Olympic level athlete, including intersectionality of identities and unique considers for treatment.
- Tailor interdisciplinary interventions for chronic pain to the unique needs of athletes.
- Acquire a framework for interprofessional collaboration between pain and sport spheres to improve outcomes for young athletes with chronic pain.

**Speaker One**

**Characterizing chronic pain in youth athletes: Unique considerations for successful rehabilitation**

Lauren Harrison

Stanford University of Medicine

**Abstract**: Sport-related injury is common in youth. While many young athletes recover and return to sport, some continue to be limited by chronic pain. Here we characterize patients with chronic pain who identified as athletes and assessed the relationship between athlete identity and pain sequelae. Youth (*N* = 305; Mage = 13.9) presenting to a multidisciplinary pain clinic completed measures assessing sport participation, athlete identity, pain-related distress, and impairment. Overall, 83.6% of participants identified as athletes (39.2% currently involved, 53.3% intending to return). A moderate level of functional impairment was observed, with those currently participating in sport reporting less impairment than those intending to return (*F =* 6.0, *p* < .05). High levels of pain catastrophizing were also reported. Athlete identity was significantly related to participant pain duration (*r* = 0.22, *p* < .01) and pain catastrophizing (*r* = 0.14, *p* < .05). Results highlight substantial overlap between chronic pain and sport, however conceptualization of chronic pain in the sport sphere often results in a biomedical approach to pain management and suboptimal outcomes. This further highlights the need for interdisciplinary pain care and interprofessional collaboration across pain providers (psychologist, physiotherapist) and sport professionals. This lecture will further contextualize these data through presentation of an athlete case study and discuss potential implications for targeting chronic pain in athletes.

**Speaker Two**

**Application of rehabilitative approaches to chronic pain in young athletes**

Karen Hurtubise

The University of Sherbrooke; Can Child Centre for Childhood Disability Research

**Abstract**: Injury and pain in sport is common and has been associated with decreases in physical functioning, social functioning, and global health-related quality of life. While many young athletes recover and return to sport, a significant proportion continues to be impacted by their injury resulting in chronic pain (CP) concerns, and at times, early departure from sport. To mitigate undesirable outcomes, interdisciplinary approaches have been established as the gold standard. However, a major barrier to physical rehabilitation for youth with CP is fear of pain and fear of movement. These fears can lead to sedentary behaviour which can thereby cause tissue changes that further increases pain and disability. Physical rehabilitation for chronic pain, which aims to reduce fear of pain and movement, tissue changes, and increase engagement in functional activities (e.g. competitive sport) differs from traditional approaches for acute pain. In this session, three specific differences will be explored and described as they relate to a young athletes with chronic pain lived experience: (1) The prioritization of active treatment strategies (e.g., graded exercise) (2) The use of psychologically informed physical treatments, more specifically goal-setting, and (3) The inclusion of sport-related activities into physical rehabilitation and graded return and exposure to the sport.

**Speaker Three**

**The importance of interprofessional collaboration for treating chronic pain in young athletes**

Courtney Hess

Stanford University School of Medicine

**Abstract**: A growing understanding of chronic pain has prompted the reconceptualization of the pain model from a biomedical to a biopsychosocial paradigm. Consistent with this paradigm shift, approaches to pediatric pain management have shifted and calls in the literature have been consistent in the need for multimodal interventions (e.g., physical, psychological) that demand interprofessional collaboration. These same calls for interprofessional collaboration have emerged in the context of athletic injury; however, researchers have pointed toward the gaps in the translation of theoretical conceptualization to professional practice which may result in a biomedical approach to pain management within the sport context. This lecture will present qualitative data from a study of an interprofessional team supporting injury rehabilitation leading into the 2014 Olympic Winter Games, highlighting factors that impacted interdisciplinary work in an elite sport context (e.g., sociocultural context, interpersonal challenges). Alongside these data, this lecture will include the experiences of an athlete who managed chronic pain within a high-intensity sport context. Data will be presented through an established team science framework, offering a conceptual frame for establishing and improving integrated treatment approaches for chronic pain with young athletes. We will conclude with a discussion of the unique barriers and catalysts to facilitating integrated work in the sport context and highlight areas for future team science research within this sphere.

**Placebo effects with treatments for pain - making much out of nothing at all?**

**Session Chair:** Javeria Ali Hashmi, PhD, Dalhousie University.

**Abstract**: Placebo effects in pain are now recognized as a significant contributor to the recipient reported response to therapies for pain. However, it remains unclear as to why these effects are a bigger contributor to the analgesic experience for certain individuals as compared to others. The contribution of the placebo response also varies with the type of analgesic modality – it is generally accepted that more invasive modalities confer a greater degree of placebo response. This proposed symposium will discuss critical elements of placebo mechanisms that have an impact on clinical practice of Pain Medicine and also inform the methodology of clinical trials of analgesic modalities. The first talk will explore the mechanisms in the nervous system that underlie placebo responses. The speaker will also explore use of quantitative sensory testing and neuroimaging techniques in determining the modulatory mechanisms that can contribute to the placebo effect. The second talk will include a discussion of observed placebo effects in clinical trials of pharmacologic analgesics. The speaker will share his experience of the approaches to determine placebo effects in clinical drug trials and practice of Pain Medicine. The third talk will focus on placebo effects associated with interventional pain techniques including neuromodulation. The speaker will compare placebo effects with non-invasive analgesic treatments against interventional procedures and highlight the similarities as well as unique differences in conferred placebo effects between these modalities. The three speakers will also provide the audience strategies to determine as well harness the placebo effect with pain treatments.

**At the end of this presentation, participants will be able to**:

- Describe the placebo effect and its relevance in affecting outcomes of pain treatments.
- Identify the mechanisms involved in the placebo effect and the evidence-base supporting these.
- Recognize the differential magnitude of placebo effects associated with analgesic treatments and their impact on treatment decisions and outcomes.

**Speaker One**

**Mechanisms of placebo effects in pain**

Javeria Ali Hashmi

Dalhousie University

**Abstract**: Placebo effect is a puzzling phenomenon where individuals can experience symptom relief from nonspecific treatments. These effects are observed widely in clinical trials and contribute significantly to analgesic outcomes from alternative or novel treatments. A better understanding of placebo effect can be useful for clinically harnessing the mechanisms that can endogenously confer pain relief. In addition, studying placebo effect can lead to a better understanding of what goes awry in persistent pain states. The prominent mechanisms through which the brain can result in placebo effects is through phenomenon such as expectations based on prior information, desire/motivation and pain intensity/need for pain relief. This talk will highlight brain mechanisms and pathways that play a key role in modulating pain based on expectations. Specifically, the speaker will present new evidence on how brain regions such as the dorsolateral prefrontal cortex and its downstream connections with periaqueductal gray area and accumbens can contribute to pain modulation. These pathways and how they may serve as useful targets for neuromodulation therapies will be examined. In addition, how psychological states such as anxiety, chronic back pain intensity and cognitive deficits in working memory can affect the pain modulating pathways will also be shown. Thus, brain mechanisms and the psychological and clinical factors that shape the endogenous aspects of treatment response will be discussed.

**Speaker Two**

**Placebo effects in randomized clinical trials of analgesics - a dirty secret or a secret friend?**

John Douglas Markman

University of Rochester School of Medicine and Dentistry

**Abstract**: In recent years, randomized clinical trials of analgesic drugs have increasingly failed to show statistically significant superiority to placebo in conditions for which their efficacy was previously shown. Efforts to reduce placebo group improvement assume that responses to active and placebo treatments are not additive and that specific methodologic features of an RCT potentially have different effects on subjects administered placebo as compared to those exposed to active analgesic treatment. Interventions to improve the ability of an RCT to demonstrate the benefits of efficacious treatment, so called “assay sensitivity,” are now routinely incorporated in phase 2 and 3 studies. This presentation will examine the study design features, site factors, and outcome measurement methods known to affect assay sensitivity of RCTs for drugs. This presentation will consider important questions such as: “Does subject training in expectation management and pain rating improve the likelihood of a positive clinical trial? Does excluding certain subjects with certain baseline pain features (e.g. extreme variability of baseline pain scores, poor diary compliance) compromise external validity of study results? Drawing on examples from recent clinical trials and analgesic drug development programs, the rationale for methodologic changes that aim to reduce nonspecific treatment effects and amplify the signal of on target analgesic benefit will be explored.

**Speaker Three**

**Interventional pain procedures and placebo effects - is the ceremony more important than the actual treatment?**

Anuj Bhatia

University of Rochester School of Medicine and Dentistry

**Abstract**: Interventional treatments for pain include procedures such as perineural injections (e.g., epidural procedures), radiofrequency ablation of innervation to bony joints, and neuromodulatory implants. Though there is a growing body of literature that is helping define the impact of these procedures, selecting appropriate recipients has been recognized as an important step prior to the procedure. Some patients with pain may perceive significant placebo effects due to the setup around the intervention. The nature of the intervention, its cost, and the proceduralist’s stature may all play in a role in enhancing the placebo effect with more invasive and expensive procedures performed by senior personnel having the greatest placebo effect. Further, higher pain intensity at baseline results in a greater placebo effect. Interestingly, when the experimental treatments were associated with better results, a greater analgesic benefit was also found for the placebo arm. This talk will summarize the evidence for placebo effects in trials of interventional procedures and it will also provide strategies for recognizing this component of the analgesic response and for factoring it in trial design. The speaker, an interventional pain physician and a clinical epidemiologist with over 100 publications, will present data from his Institution on placebo effects measured during trials of neuromodulatory implants for pain. Issues around appropriate blinding of participants, proceduralists, and outcome assessors to reduce the placebo effect will also be discussed.

**Concurrent Session Seven**

**Prenatal and neonatal exposure to pain and its long-term effects on the developing brain**

**Session Chair:** Jillian Vinall Miller, PhD, University of Calgary.

**Session Abstract**: The third trimester represents a critical period of fetal brain development. Maternal pain-related stress during this period, may alter thalamocortical connections, thereby leading to heightened stress-related behavior during childhood. In children born very preterm, repeated exposure to neonatal pain robustly predicts alterations in brain connectivity and is associated with greater cognitive and behavioral problems in early childhood, which often persist into adulthood. Animal models are providing greater insights into the mechanisms underlying the relationships between early life exposure to pain, and the corresponding cellular and molecular changes in the CNS, which are driving long-term changes in behavior. In this symposium, the implications of both prenatal and neonatal exposures to pain will be presented and discussed. Jenna Jessa (MSc Student in Medical Sciences, University of Calgary) will present findings from her Master’s Thesis, examining maternal pain trajectories through the perinatal period and into the postpartum. Dr. Emma Duerden (Assistant Professor, Western University) will present her work on early procedural pain and altered thalamic development, with a focus on multimodal neuroimaging techniques and behavioural assessments to inform outcome prediction and precision medicine. Finally, Dr. Simon Beggs (Associate Professor, University College London) will bridge the gap between preclinical and clinical research by sharing his work on the long-term effects of early life exposure to pain. Across all three speakers, it will be clear that the negative consequences of unmanaged pain both in mothers and infants during the 3rd trimester, or during a developmentally equivalent period in rodents, are significant, persistent, and preventable.

**At the end of this presentation, participants will be able to**:

- Describe trajectories of pain intensity, pain catastrophizing and pain interference during the perinatal period and, the possible implications of trajectory membership for both the mothers and infants.
- Identify the motivation and methodologies for studying early brain development and outcomes in high-risk infants, with an emphasis on risk factors for altered brain development.
- Demonstrate how complementary methodologies between preclinical and clinical research can be used to mechanistically understand the lasting impact of early life pain.

**Speaker One**

**Pain trajectories in the perinatal period and postpartum**

Jenna Jessa

University of Calgary

**Abstract**: The rapid hormonal and physiological changes occurring during a normal pregnancy give rise to recurrent, and sometimes constant, pain for women. Women with worse pain symptoms are more likely to report symptoms of anxiety, depression, and/or insomnia during the perinatal period, which may have implications on their labour and delivery. Children of parents with chronic pain are at risk for poorer physical health, increased chronic pain incidence and worsening psychological and functioning outcomes. Given the burden of pain during pregnancy, we sought to identify trajectories of pain symptomology from early pregnancy to postpartum. Pregnant women completed measures of pain catastrophizing, pain intensity, pain interference, insomnia symptoms, depression, and generalized anxiety at four time-points. Group-based trajectory analysis was utilized to determine trajectories of pain intensity, pain catastrophizing and pain interference. A one-class pain intensity model, two-class pain catastrophizing model, and three-class pain interference model were identified. Adaptive lasso and imputation allowed analysis of model validity. Significant individual predictors of trajectory membership included symptoms of anxiety and depression, insomnia, and baseline pain symptomology. These findings may help to identify women who are at high risk for experiencing pain symptoms and could aid in developing targeted treatment strategies to prevent mothers and their children from the consequences of ongoing and repeated exposure to pain.

**Speaker Two**

**Early procedural pain, thalamic development and functional outcomes in very preterm born neonates**

Emma Duerden

Western University

**Abstract**: Very preterm neonates (born <32 weeks’ gestation [GA]) can often spend the first few weeks of life in intensive care. Many of these babies require life-saving measures that often exposes these tiny neonates to hundreds of invasive procedures such as heel lances, endotracheal intubations, and surgical interventions. Increasing evidence suggests that exposure to noxious procedures early in life is associated with delayed brain development and adverse cognitive, motor and sensory outcomes. Further, clinical investigations and animal models have indicated later atypical pain-related behaviours and stress responses may be more pronounced if experienced at earlier gestational ages. The immature nervous system, particularly thalamocortical connections, may show enhanced vulnerability to excessive sensory stimulation, and may contribute to widespread alterations in brain function and subsequent behaviour. In this symposium presentation, recent research in humans demonstrating an association among invasive procedures and altered thalamocortical development will be presented. These alterations in thalamocortical development will be linked with developmental outcomes. Research findings will be discussed in relation to individual differences in neonatal patients, which will provide the basis for developing future clinical strategies that may inform individualized care, treatment responses, outcomes and as well as selection for future clinical trials.

**Speaker Three**

**Long-term consequences of pain in early life**

Simon Beggs

University College London

**Abstract**: Pre-term infants are exposed to tissue-damaging and potentially painful procedures as part of their routine care. Furthermore, complications associated with prematurity lead to an increased requirement for surgical intervention in this patient population. Early life surgery is an identified risk factor for changes in future pain responses. What are the neurobiological factors that drive the long-term effects of these events? While human studies are revealing the changes in neural development that ensue from early life pain, and improving the prediction of vulnerability to persistent post-surgical pain, parallel translational pre-clinical evaluations are required to reveal the underlying cellular and molecular mechanisms involved, specifically age- and sex-dependent influences. It is now clear that pain in early life impacts circuits that contribute to sensory, discriminatory, emotional, and cognitive behaviours; and progress in our understanding has been limited by tools that fail to encompass these disparate effects. Furthermore, these effects are mediated throughout the nociceptive pathway, and may be modulated by several mechanisms such as ongoing excitation, decreased inhibition, and failure of active resolution mechanisms. Pre-clinical methodologies are generally either ex-vivo, and hence unable to make predictions over time, or in vivo, but focussed on discrete brain regions, and therefore unable to make predictions across space. This presentation will discuss how we can close the gap between preclinical and clinical research by using complementary methodologies to access mechanistic understanding of the influence of early life pain and/or injury on pain behaviour in later life.

**Implantable neuromodulation therapies for pain - as simple as buzzing the pain away or more to it than meets the eye?**

**Session Chair:** Anuj Bhatia, MD PhD FRCPC (Anaesthesia, Pain Medicine), University of Toronto and University Health Network - Toronto Western Hospital.

**Session Abstract**: The treatment of pain remains a public health concern with a growing cohort plagued by medically refractory, unrelenting severe pain that ruins their quality of life and productivity. Neuromodulation is defined as the alteration of nerve activity through targeted delivery of a stimulus, such as electrical stimulation or chemical agents, to specific neurological sites in the body. Though the first neuromodulation implant was performed in 1967, it has received more attention in the last decade and it is an expanding therapy that incorporates an array of minimally invasive, and surgical electrical therapies. In this symposium, we will focus on the following implantable therapies for refractory pain – deep brain and motor cortex stimulation (DBS, MCS), spinal cord stimulation (SCS), peripheral nerve stimulation (PNS), and intrathecal targeted drug delivery (IDD). These therapies share some common features – appropriate selection of individuals likely to benefit, the option of a pre-implant trial, equipment consisting of electrical leads, catheters, internal pulse generators, pumps, surgical considerations, and periodic long-term follow-up. The analgesic impact of these therapies including its magnitude and persistence will be presented at the symposium along with implications for cost and adverse effects. Also, we will present evidence for these therapies that is at different stages - early for DBS, MCS and PNS while developed and robust for SCS and TDD. The four speakers including a person with lived experience of pain and neuromodulation will address different aspects of this topic. Areas needing research in this field will be discussed in this symposium.

**At the end of this presentation, participants will be able to**:

- Recognize the types of neuromodulation therapies and their application in relieving pain.
- Identify the indications for neuromodulation for treating common refractory pain syndromes and the evidence-base supporting these.
- Appraise the limitations that impact on the use of neuromodulation for pain including gaps in awareness of healthcare providers and support from healthcare payors.

**Speaker One**

**Brain and peripheral neuromodulation for pain**

Keith Mc Dougall

Western University; London Health Sciences Centre

**Abstract**: Deep brain stimulation (DBS) and motor cortex stimulation (MCS) have been used for treating neuropathic pain for over 30 years. DBS has been used to stimulate a variety of brain targets for pain relief including the sensory thalamus (the majority of reports), periaqueductal grey or periventricular area, and the anterior cingulate cortex. DBS can be used for neuropathic pain of spinal origin and poststroke pain. MCS is used for thalamic pain, phantom limb pain, postherpetic neuralgia, brachial plexus avulsion, poststroke pain, complex regional pain syndrome, pain secondary to multiple sclerosis, spinal cord injury pain, and posttraumatic brain injury pain. However, there is ongoing research on patient selection, optimal stimulation parameters, and long-term outcomes of both modalities. Peripheral nerve stimulation (PNS) has recently seen a resurgence of interest with customized neuromodulation systems. PNS is used to treat occipital neuralgia, peripheral mononeuropathies, and for field stimulation for somatic abdominal wall pain. This presentation will discuss the indications, equipment, long-term management, complications, and areas needing research for DBS, MCS, and PNS. Speaker: Dr. Keith MacDougall iss is the current President of the Canadian Neuromodulation Society and his ongoing research interests include the effectiveness of neuromodulation for the treatment of chronic neuropathic pain and headaches and the application of novel stimulation techniques.

**Speaker Two**

**Intrathecal drug delivery for pain - targeted and effective?**

Jill Osborn

University of British Columbia; St. Paul’s Hospital, Vancouver, BC

**Abstract**: Intrathecal targeted Drug delivery (TDD) was first used in the 1980s to treat intractable pain. It is now utilised to relieve both malignant and non-malignant pain by targeting pain receptors at the spinal level thus reducing the sedating effects at the brain level. Though initial research on the effectiveness of this therapy was published 20 years back, recent research has highlighted the cost-savings to the healthcare system, reduction in the use of opioids, increase in survival in patients with cancer-related pain, and improvement in the quality of life with TDD. This presentation will highlight the indications, important aspects of long-term management, and potential complications of TDD. Recent guidelines from the Polyanalgesisc Consensus Conference on combining different types of analgesics (local anesthetics, opioids, alpha-2 agonists, and others) will also be highlighted with a focus on regimens that are feasible in the Canadian healthcare systems.

**Driving policy development for pain in Canada: Perspectives from a governmental, lived experience, clinician, and advocacy group lens**

**Session Chair:** Fiona Campbell, MD, Sick Kids Hospital, University of Toronto.

**Session Abstract**: The Canadian Pain Task Force provided its final report, An Action Plan for Pain in Canada in March 2021. This report provided over 150 recommendations on priority actions, so that people with pain are recognized and supported and that pain is understood, prevented, and effectively treated across Canada.

Over the years, people living with pain, health care professionals, researchers, policy makers, and non-governmental organizations have been contributing to a movement for action on pain in Canada. This movement is gaining momentum, and these diverse perspectives are important to deliver effective policies that will help address this complex issue.

In this session, multiple perspectives on enacting policy changes at the federal, provincial and territorial levels to enhance pain treatment and management in Canada will be shared. Panel members will discuss successes they have had in driving policy changes for pain management, and discuss possible future opportunities for change. They will also discuss any challenges or roadblocks they have encountered in their work. By showing various perspectives, attendees will be able to see how progress can be made at different levels and from different groups.

Panel members will include a representative from the federal government (Jean-François Leroux, Health Canada), a representative from a provincial government (Tracy Wasylak, Alberta Health Services), a person living with pain (Jacques Laliberté, AQDC), a clinician (Dr. Fiona Campbell, SickKids), and a representative leading a national pain initiative (Maria Hudspith, Pain Canada).

**At the end of this presentation, participants will be able to**:

- Describe the roles and responsibilities of different stakeholders in driving policy changes in pain-related areas.
- Learners will be able to describe challenges and limitations in the development implementation of pain policies at the national, federal and provincial/territorial levels.
- Recognize progress in advancing pain policies at the national, federal and provincial/territorial levels.

**Speaker One**

**Driving policy development for pain: Perspective from a person living with pain involved in development of pain policies at the federal and provincial level**

Jacques Laliberté

Association de la douleur chronique du Québec

**Abstract**: In its final report, the Canadian Pain Task Force highlighted the importance for people with lived experience to be engaged in national mechanisms to support the implementation of the Action Plan on Pain in Canada. As a founding member of the *Association Québécoise de la douleur chronique* and a former member of the Canadian Pain Task Force, Jacques will speak to his experience in the development of pain policies at the federal and provincial level and the importance of involving people living with pain in the development and implementation of pain policies.

**Speaker Two**

**Driving policy change for pain through the Pain Canada initiative**

Maria Hudspith

Pain BC

**Abstract**: Pain Canada is a multi-stakeholder national action network created in response to the Canadian Pain Task Force’s final report “An Action Plan for Pain in Canada”. The Action Plan called for national mechanisms to improve coordination, create community capacity, develop and disseminate pain-related guidance and best practices and to enable collaboration among governments, regulators and health professional organizations, people with lived experience, non-governmental organizations, researchers, employers, and other stakeholders with a role to play in the Action Plan’s implementation. Maria Hudspith, Executive Director of Pain BC, will discuss the progress that Pain Canada has made to support and empower people living with pain, expand access to clinical education, foster patientoriented pain research and knowledge mobilization, as well as combat stigma and raise awareness. She will also discuss the challenges that Pain Canada has faced in working towards these goals, the myriad opportunities presented by the Pain Canada initiative, and how non-profit organizations can help drive policy changes at the federal, provincial and territorial levels.

**Speaker Three**

**Driving policy development for pain: Supporting the development and implementation of Alberta’s pain strategy**

Tracy Wasylak

Alberta Health Services

**Abstract**: In 2019, the Province of Alberta launched the Alberta Pain Strategy (2019-2024), which outlined a coordinated, provincial approach to help Albertans manage pain across their lifespan. This Strategy was developed by a multi-stakeholder group led by Alberta Health Services’ Strategic Clinical Networks™ (SCNs) and the Pain Society of Alberta. More than 360 healthcare providers, administrators, researchers, patients and families from across Alberta came together and contributed to the development of the strategy. Over the past three years, substantial work has been undertaken to implement the actions within the strategy. Tracy Wasylak will discuss enablers that contributed to support the development of the strategy, advancements towards its implementation and progress to date; including the development of a provincial pain program.

**Perceived injustice and pain: Causes, consequences and mechanisms of action**

**Session Chair:** Michael Sullivan, PhD, McGill University.

**Session Abstract**: For many individuals, life following work injury will be characterized by persistent physical and emotional suffering. Clinical anecdotes abound of persistent pain sufferers’ reflections on the ‘injustice’ of living with ongoing pain and disability. Recent research has shown that perceptions of injustice following debilitating injury can adversely impact on recovery trajectories. High levels of perceived injustice have been linked prospectively with more intense pain and emotional distress, the persistence of symptoms of depression and post-traumatic stress symptoms, and prolonged work disability. This symposium will summarize the results of recent studies examining 1) the health and mental health consequences of perceived injustice, 2) the physical and psychological determinants of perceived injustice in individuals with debilitating pain conditions, and 3) injured individuals’ accounts of the factors that have led them to experience their post-injury life as unjust. Possible pathways by which perceived injustice impacts on recovery processes will be addressed. The presentations in this symposium will highlight how the identification of the sources of perceived injustice will be critical to the development of avenues of intervention aimed at fostering more favourable recovery following debilitating injury. It will be argued that a paradigm shift in approaches to evaluation and treatment might be required in order to yield meaningful reductions in perceived injustice. Such a paradigm shift might entail broadening the targets of assessment and intervention beyond the ‘perceptions’ of the injured individual to include potential external sources of injustice (e.g., employer, insurer, health care provider) in the ‘treatment plan.’

**At the end of this presentation, participants will be able to**:

- Familiarize themselves with what is known about the consequences of perceived injustice associated with pain.
- Explore research findings addressing the determinant of perceived injustice in individuals with disabling musculoskeletal injuries.
- Recognize injured workers’ perspectives on sources of injustice in the immediate aftermath of work injury.

**Speaker One**

**Injured workers’ perspectives on the sources of perceived injustice following debilitating injury**

Heather Adams

School of Social Work, Halifax

**Abstract**: Using a qualitative approach, participants with elevated scores on a measure of perceived injustice (n = 30) were interviewed about the factors that contributed to their sense of injustice following a disabling work injury. A grounded theory methodology was used to examine the broad classes of injusticeeliciting situations or events that participants experienced as unjust in the weeks following occupational injury. Three dominant themes emerged from the interviews: 1) Invalidation, 2) Undeserved suffering and 3) Blame. Participants described experiences invalidating communication from employers, health professionals and insurance representatives. Participants’ interview responses also highlighted the sense of injustice resulting from the interference of pain with participation in valued life activities, and the expectation of prolonged or permanent pain. Blame attributions were made for experiences that contributed to the participants’ suffering as a result of lack of understanding, lack of acknowledgement or denial of the participant’s condition. The findings not only advance knowledge about the factors contributing to the emergence of perceived injustice in the aftermath of disabling injury, but they also point to possible pathways of intervention that might prevent or reduce perceptions of injury following disabling injury.

**Speaker Two**

**Perceived injustice as a risk factor for pain-related outcomes in adults with persistent pain conditions**

Junie Carrière

Université de Sherbrooke

**Abstract**: This presentation will summarize the findings of recent research examining the association betweenperceived injustice and pain-related outcomes in adults with persistent pain conditions. Published evidence demonstrates that perceived injustice is associated with increased pain intensity, more pronounced and prolonged disability, and adverse mental health outcomes in individuals with persistent pain conditions. The relation between perceived injuctice and problematic recovery has been demonstrated in numerous populations including in individuals who have sustained whiplash injuries, catastrophic injuries, traumatic head injuries, individuals with work-related low back pain, fibromyalgia, sickle cell disease, osteoarthritis, rheumatoid arthritis, and Major Depressive Disorder. The presentation will highlight findings relevant to discerning the pathways by which perceptions of injustice might contribute to problematic recovery outcomes. Discussion will address the importance of addressing perceptions of injustice in the assessment and treatment of individuals with persistent pain conditions.

**Speaker Three**

**Bidirectional relations between perceived injustice and recovery outcomes in individuals with disabling musculoskeletal injuries**

Michael Sullivan

McGill University

**Abstract**: Numerous investigations have examined the relation between perceptions of injustice in individuals and recovery outcomes in individuals suffering from a wide range of debilitating health and mental health conditions. The emerging pattern of findings suggests that perceived injustice triggers a cascade of cognitive, emotional and behavioural reactions that can impede recovery. This presentation will describe the results of a prospective study examining the causes and consequences of perceived injustice in the immediate aftermath of a disabling work injury. The results of this study showed that perceptions of injustice were prevalent following musculoskeletal injury. For the majority of participants, scores on the measure of perceived injustice remained stable or decreased though the study period. Perceived injustice scores increased for 23% of the sample. Cross-sectional regression analyses revealed that scores on measures of pain and depression contributed significant unique variance to the prediction of perceived injustice scores. The results of cross-lagged regressions suggested bi-directional influences between perceived injustice, pain and depression. Early changes in perceived injustice predicted subsequent changes in pain and depression, and early changes in pain and depression predicted subsequent changes in perceived injustice. The findings of the study point to the importance of early screening of perceptions of injustice in individuals with disabling work injuries and call for the development of interventions specifically designed to prevent or reduce perceptions of injustice.

